# Gene expression profiling of spontaneously occurring canine mammary tumours: Insight into gene networks and pathways linked to cancer pathogenesis

**DOI:** 10.1371/journal.pone.0208656

**Published:** 2018-12-05

**Authors:** Shahid Hussain, Sonal Saxena, Sameer Shrivastava, Ashok Kumar Mohanty, Sudarshan Kumar, Rajkumar James Singh, Abhinav Kumar, Sajad Ahmad Wani, Ravi Kumar Gandham, Naveen Kumar, Anil Kumar Sharma, Ashok Kumar Tiwari, Raj Kumar Singh

**Affiliations:** 1 Division of Veterinary Biotechnology, ICAR-Indian Veterinary Research Institute [Deemed University], Izatnagar, Bareilly, UP, India; 2 Animal Biotechnology Division, ICAR-National Dairy Research Institute [Deemed University], Karnal, Haryana, India; 3 Department of Computer Science and Engineering, Indian Institute of Technology (IIT) BHU, Varanasi, India; 4 The Ohio State University, Columbus, Ohio, United States; 5 National Institute of Animal Biotechnology, Miyapur, Hyderabad, Telangana, India; 6 Division of Veterinary Surgery, ICAR-Indian Veterinary Research Institute [Deemed University], Izatnagar, Bareilly, UP, India; 7 Division of Veterinary Pathology, ICAR-Indian Veterinary Research Institute [Deemed University], Izatnagar, Bareilly, UP, India; University of South Alabama Mitchell Cancer Institute, UNITED STATES

## Abstract

Spontaneously occurring canine mammary tumours (CMTs) are the most common neoplasms of unspayed female dogs leading to thrice higher mortality rates than human breast cancer. These are also attractive models for human breast cancer studies owing to clinical and molecular similarities. Thus, they are important candidates for biomarker studies and understanding cancer pathobiology. The study was designed to explore underlying molecular networks and pathways in CMTs for deciphering new prognostic factors and therapeutic targets. To gain an insight into various pathways and networks associated with the development and pathogenesis of CMTs, comparative cDNA microarray expression profiling was performed using CMT tissues and healthy mammary gland tissues. Upon analysis, 1700 and 1287 differentially expressed genes (DEGs, *P ≤* 0.05) were identified in malignant and benign tissues, respectively. DEGs identified from microarray analysis were further annotated using the Ingenuity Systems Pathway Analysis (IPA) tool for detection of deregulated canonical pathways, upstream regulators, and networks associated with malignant, as well as, benign disease. Top scoring key networks in benign and malignant mammary tumours were having central nodes of VEGF and BUB1B, respectively. Cyclins & cell cycle regulation and TREM1 signalling were amongst the top activated canonical pathways in CMTs. Other cancer related significant pathways like apoptosis signalling, dendritic cell maturation, DNA recombination and repair, Wnt/β-catenin signalling, etc. were also found to be altered. Furthermore, seven proteins (ANXA2, APOCII, CDK6, GATC, GDI2, GNAQ and MYH9) highly up-regulated in malignant tissues were identified by two-dimensional gel electrophoresis (2DE) and MALDI-TOF PMF studies which were in concordance with microarray data. Thus, the study has uncovered ample number of candidate genes associated with CMTs which need to be further validated as therapeutic targets and prognostic markers.

## Introduction

Spontaneously occurring mammary tumours in dog have been demonstrated as useful models for human breast cancer studies owing to their similarities in histological features and disease biology; associated risk factors, clinical progression and response to treatment; biomarkers and molecular targets etc. [1–2]. Although widely used, xenogeneic, as well as, transgenic mouse tumour models fail to mimic various features of human breast cancers like steroid hormone dependency, tumour microenvironment, heterogeneous behaviour etc. [1–2]. With complete sequencing of dog genome revealing close similarities between dog and humans at genetic level, spontaneously occurring canine mammary tumours (CMTs) have emerged as attractive alternatives to artificially induced tumours in mice.

CMTs account for approximately 50% of all tumours of female dogs with approximately 50% cases being malignant in nature [3–4]. Dogs with poorly differentiated tumours have increased risk of recurrent or metastatic disease, with 90% recurrence rate for the most dedifferentiated tumours [4]. In unspayed female dogs, CMTs are the most common neoplasms leading to at least three times higher mortality rates than human breast cancer [5]. Presently, the genes accountable for the aggressive behaviour of mammary cancer are not very clear, and poor prognosis associated with malignant mammary cancers emphasize the necessity to unravel the underlying pathways and genes which could act as targets for therapy. The molecular mechanisms, networks and pathways contributing to the biological behaviour of CMTs are poorly understood and there is a lack of knowledge about reliable tumour markers [6]. The detailed characterization of the dysregulated genes and careful mining of the gene networks and pathways could also be of great help in identification of diagnostic, as well as, prognostic biomarkers.

The study was designed with the objective of identification of new prognostic factors and targets for therapy, as well as, underlying pathological mechanisms and networks. To have an insight into various pathways and networks associated with development and pathogenesis of CMTs, comparative cDNA microarray analysis was performed. The gene expression profiles of malignant and benign CMT tissues were compared with healthy mammary tissues. To gain a deeper knowledge, differentially expressed genes (DEGs), identified from this analysis were further subjected to functional annotation using the Ingenuity Systems Pathway Analysis (IPA) tool. IPA was used to detect upstream regulators, pathways and networks associated with CMTs. The study has uncovered numerous candidate genes involved in pathogenesis of CMTs, which need to be further validated as therapeutic targets and prognostic markers for mammary cancer.

## Materials and methods

**Fig 1:**
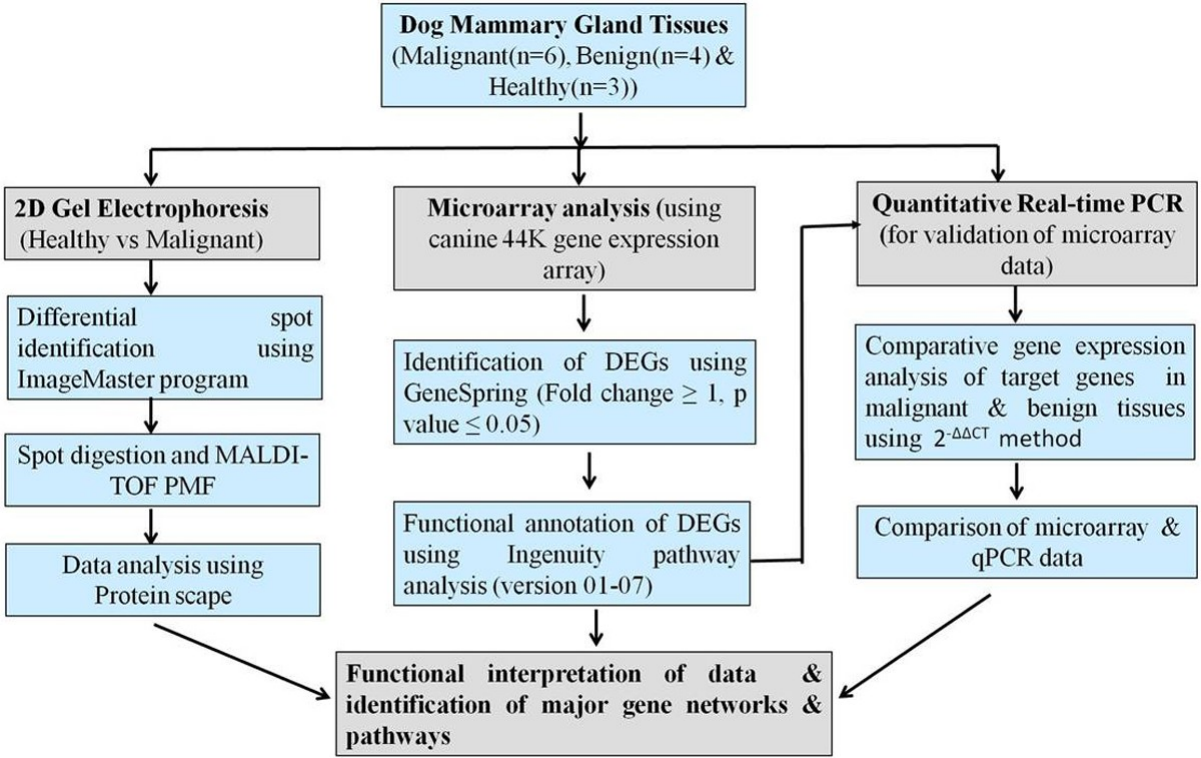
The overview of the methodology used for gene expression profiling and identification of major networks and pathways associated with canine mammary tumour (CMT).

The overview of the methodology used for gene expression profiling of CMTs is depicted in Fig 1.

### Tissue samples

All experimental procedures involving animals were in accordance with Breeding of and Experiments on Animals (Control and Supervision) Amendment Rules, Government of India, 2005 and were approved by Institute Animal Ethics Committee (IAEC) of ICAR-Indian Veterinary Research Institute (ICAR-IVRI), Izatnagar. Cancer tissue samples (n = 10) were collected from clinical cases of CMTs which were referred for surgery to ‘Referral Veterinary Polyclinic’, ICAR-IVRI, Izatnagar. Reference healthy tissues (n = 3) were collected from healthy dogs with prior written consent from the owner. Animals were given local anaesthetic (1% lignocaine) prior to tissue collection. Tissue sections were collected immediately after surgical resection and stored in RNAlater at -80°C for RNA isolation. Part of tissue section was also preserved in neutral buffered formalin for histopathology. For proteomic studies, tissues were preserved at -80°C.

### Histopathological classification

For histopathology, formalin-fixed tissues were paraffin-embedded and cut into 5 μm sections. Sections were then mounted on 3-aminopropyl-triethoxy-silane (APTES) coated slides, and air-dried overnight at 37°C. Prepared slides were deparaffinized in three washes of xylene (10 min each), and rehydrated in graded concentrations of ethanol. Slides were then stained with Eosin-Hematoxylin and studied for identification for tumour type and grade. Histopathological classification of the paraffin embedded tissue sections was done as per Goldschmidt *et al*. [7]. The tumours of mammary gland used in the current study were classified into malignant (n = 6) and benign (n = 4) nature. The malignant tumours were classified histopathologically as tubuloacinar solid carcinoma, papillary and squamous cell mixed carcinoma, malignant myoepithelioma, squamous cell carcinoma, lipid rich carcinoma and fibrosarcoma. Benign tumours were classified histopathologically as adenomas, papillary adenoma and cystadenomyoepithelioma. Histological grades and types of cancer tissues used in study are provided in supplementary information. Healthy mammary tissue samples (n = 3) were also examined histologically for confirming absence of malignancy and any other pathological condition. Histopathological details of tumour tissues used for the study are provided in S1 Table.

### Isolation of total RNA

Total RNA was isolated from frozen tissue samples preserved in RNAlater using QIAGEN RNeasy microarray tissue mini kit as per the manufacturer’s protocol. The purity and concentration of total RNA extracted was checked spectrophotometrically. The quality check of the isolated RNA was performed in Agilent 2100 bioanalyzer as per the manufacturer’s recommendations using the Agilent RNA 6000 Nano Kit. RNA samples having RNA integrity number (RIN) greater than 7 were used for further studies.

### Microarray hybridization and data analysis

For performing microarray studies, 200ng of RNA was reverse transcribed into cDNA using LowInput QuickAmp labelling kit (Agilent, USA) and further converted into cRNA to cause incorporation of nucleotides containing cyanine 3 (Cy-3-dCTP) as per the manufacturer’s instructions. Labelled cRNA was purified using QIAquick purification columns. The quality and concentration of the labelled cRNA was checked spectrophotometrically and hybridization experiments were performed at 65°C for 17 hours on canine (v2) gene expression 44K array comprising of 43803 canine probes (Agilent Technologies, Cat No. G2519F-021193). Each sample was hybridized to set of duplicate arrays. After generating the microarray scan images, the signal intensities were extracted using feature extraction software (version 10.7.3). The quality of hybridization and overall chip performance was monitored by visual inspection of both internal quality control checks and the raw scanned data. The data generated after feature extraction was exported into GeneSpring software (version 13.0) to identify the differentially expressed genes (DEGs) between malignant v/s healthy and benign v/s healthy. Mean normalised signals from malignant (n = 6), benign (n = 4) and healthy (n = 3) tissues were clubbed as a single experimental group and named as malignant, benign, and healthy respectively and used for comparative expression analysis. Paired t-test using storey with bootstrapping correction (P value ≤ 0.05) was used to identify differentially expressed mRNAs between malignant and benign tumours with respect to healthy mammary tissue. The gene expression fold change between tumour and healthy tissue was calculated by comparison of the normalised signal intensity values. Data was submitted to NCBI GEO database (Accession number GSE104733).

### Quantitative real time PCR (qPCR)

To verify the microarray gene expression data, 14 genes were selected for validation using qPCR. The genes selected for validation of microarray results included the top up-regulated genes, some randomly selected genes, as well as, genes having potential role in cancer pathogenesis. These genes included *MMP9*, *CHI3L1*, *BIRC5*, *BIRC2*, *TLR2*, *CTSS*, *PGAM1*, *KIF11*, *COL11A1*, *SRFP2*, *TOP2A*, *CPA2KL*, *CDCA3*, *RAB31* genes. The primers were designed for qRT-PCR analysis using the Integrated DNA technologies-PrimerQuest Tool. The details of primers sequences used for the study are mentioned in S2 Table. The cDNA was synthesized using Revert Aid First Strand cDNA synthesis kit (Thermofischer Scientific, USA) according to the manufacturer’s instructions and qRT-PCR was performed using Applied Biosystems 7500 Fast system using 2X SYBR Green Master mix (Sigma Aldrich, USA). Gene expression in each sample was normalized against the expression of housekeeping gene (β-actin). The relative expression of each sample was calculated using the 2^−ΔΔCT^ method with healthy mammary tissue as calibrator and log_2_ fold change was plotted.

### Two-dimensional gel electrophoresis (2DGE) of CMT and healthy mammary tissue

Tissue samples (~20mg) were homogenized in lysis buffer [8M urea, 2M thiourea, 4% CHAPS, 30mM Tris, pH 8.5] and kept on rotator for 15 mins followed by sonicating twice for 20s. The samples were then centrifuged at 13000 rpm for 20 mins at 4°C. Crude tissue lysate was further subjected to clean up using Ready Prep 2-D clean up kit (Bio-Rad, USA). Post clean up protein was quantified using 2-D Quant Kit (GE Healthcare, USA). Total 400μg of protein sample in 125μl destreak solution (GE healthcare, USA), containing Bio-Lyte 3/10 Ampholyte (40%) (@0.2–2% final concentration), was loaded on 7 cm ReadyStrip IPG Strip [pH 3–10] (Bio-Rad, USA). Protein was loaded onto the IPG strips by a passive rehydration method. After rehydration, isoelectric focusing (IEF) was performed on an Ettan IPGphore III apparatus at 20°C with increasing voltages 150V-200Vh, 1000V-200Vh, 5000V-4000Vh, 5000-1250Vh. The focused IPG strips were subjected to reduction with 1% w/v DTT in 10 mL of equilibration buffer (6 M urea, 50 mM Tris- HCl pH 8.8, 30% v/v glycerol, and 2% w/v SDS) followed by alkylation with 2.5% w/v iodoacetamide in the same buffer. The strips were then placed on the top of 12% resolving gels and fixed by ReadyPrep Overlay Agarose (BioRad, USA). For spot picking, gels were stained with commassie brilliant blue (R-350) stain. ImageMaster 2D Platinum v7.0 software was used for analysis and spot selection. After an automated spot detection, the protein spots were checked manually in order to eliminate any possible artifacts such as gel background or streaks and selected spots were picked. Proteins at the excised spots were then subjected to MALDI-TOF PMF analysis for their identification.

### Functional interpretation of microarray data and network analysis

To understand the functional significance of dysregulated genes in malignant and benign tumours of mammary gland, Ingenuity Pathway Analysis (IPA version 01–07) was used. Using the IPA tool, networks were generated based on an algorithmically generated score. The Z- score, a numerical value was used to rank networks according to how relevant they were to genes presented within the data set. Canonical pathways significant to the input data set were identified from the IPA library of canonical pathways based upon 2 parameters, viz., (1) The ratio of the number of genes within the data set mapping to the pathway divided by the total number of genes mapping to the canonical pathway and (2) a *P* value (calculated based upon Fischer’s exact test) determining the probability that each bio-function assigned to that dataset and the canonical pathway is not due to chance alone. Upstream regulators are defined as the genes that affects the expression of numerous other genes, while canonical pathways are the idealized or generalized pathways that represent common properties of a particular signalling module or pathway.

## Results

### Differentially expressed genes (DEGs) in canine mammary tumour tissues

**Fig 2:**
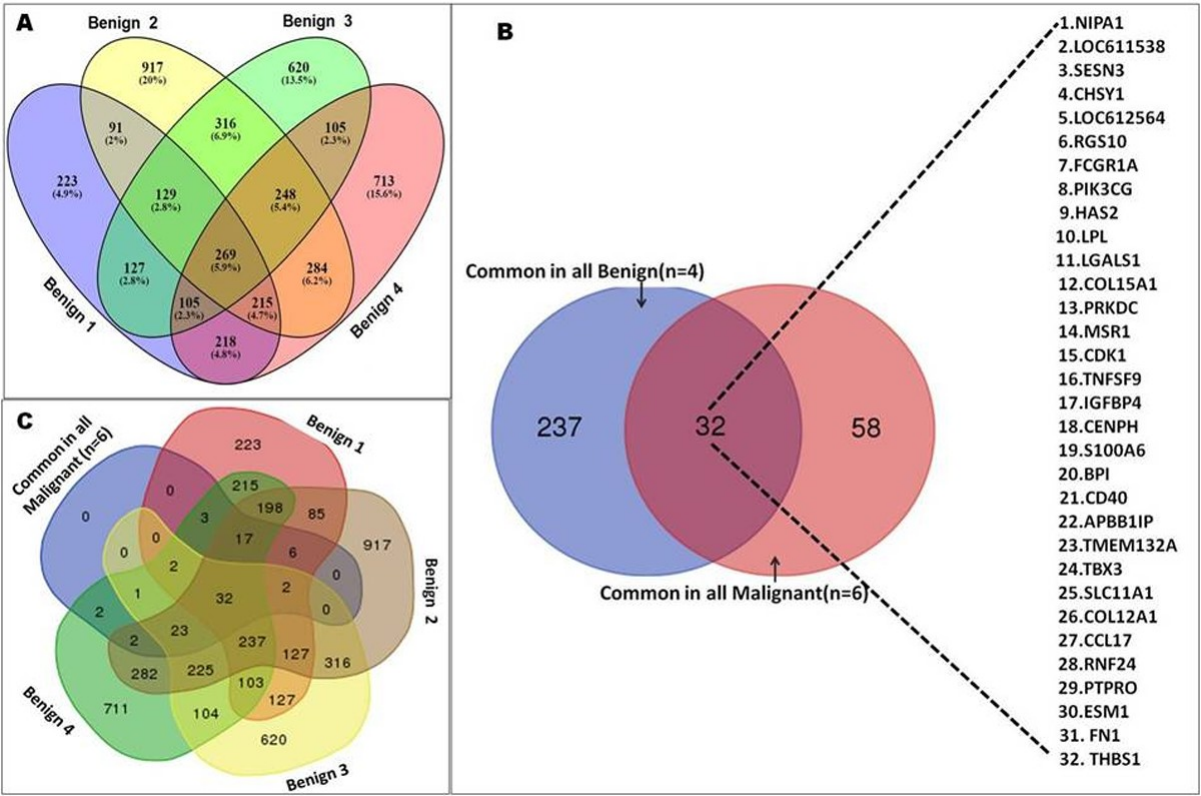
Venn diagram analysis showing overlap of dysregulated genes among malignant and benign mammary cancer tissues. (A) Overlay of up-regulated genes (logFC>1) among benign tumour tissues. (B). Overlay of genes commonly up-regulated in all the malignant and benign tissues. 269 genes were commonly up-regulated in all the benign tissues (n = 4), whereas 90 common genes were up-regulated in all the malignant tissues (n = 6).) Out of these 32 genes were commonly dysregulated in all the malignant (n = 6) and benign tissues (n = 4) studied. (C) Overlay of genes up-regulated in all the malignant tissues and individual benign tissues (1–4). Analysis revealed that there was no such gene which was uniquely up-regulated in all the malignant tissues, as the dysregulated genes which were common in all the malignant tissues were also present in either one or all the benign tissues.

To identify differentially expressed candidate genes, gene expression profiles of malignant and benign CMT tissues were comparatively analyzed with healthy mammary tissues by cDNA microarray technique. On analysis, 1700 and 1287 DEGs (*P ≤* 0.05) were identified in malignant and benign tumours of mammary gland respectively. Of these, 765 genes were up-regulated and 935 genes were down-regulated in malignant cases. Among the benign tumours, 744 genes were found to be up-regulated and 543 genes were down-regulated. Upon Venn analysis of up-regulated genes (log FC>1), it was observed that 269 genes were commonly up-regulated in all the benign tissues (Fig 2A), whereas 90 common genes were up-regulated in all the malignant tissues. Out of these 32 genes, displayed in Fig 2B, were commonly dysregulated in all the malignant and benign tissues studied. However, there was no such gene which was uniquely up-regulated in all the malignant tissues, as the dysregulated genes which were common in all the malignant tissues were also present in either one or all the benign tissues (Fig 2C). Among malignant tumours, maximum fold change was observed for *COL11A1* gene (Log_2_ FC = 4.8), while among benign tumours maximum fold change was observed with MMP3 gene (Log_2_ FC = 6.6). Top five up-regulated genes in malignant tumours were *COL11A1*, *SFRP2*, *LCN2*, *COL2A1 and H19*, while top up-regulated genes in benign tumours were *MMP3*, *MMP1*, *AREG*, *PTHLH* and *SFRP2*.

### Validation of DEGs by real time PCR

**Fig 3:**
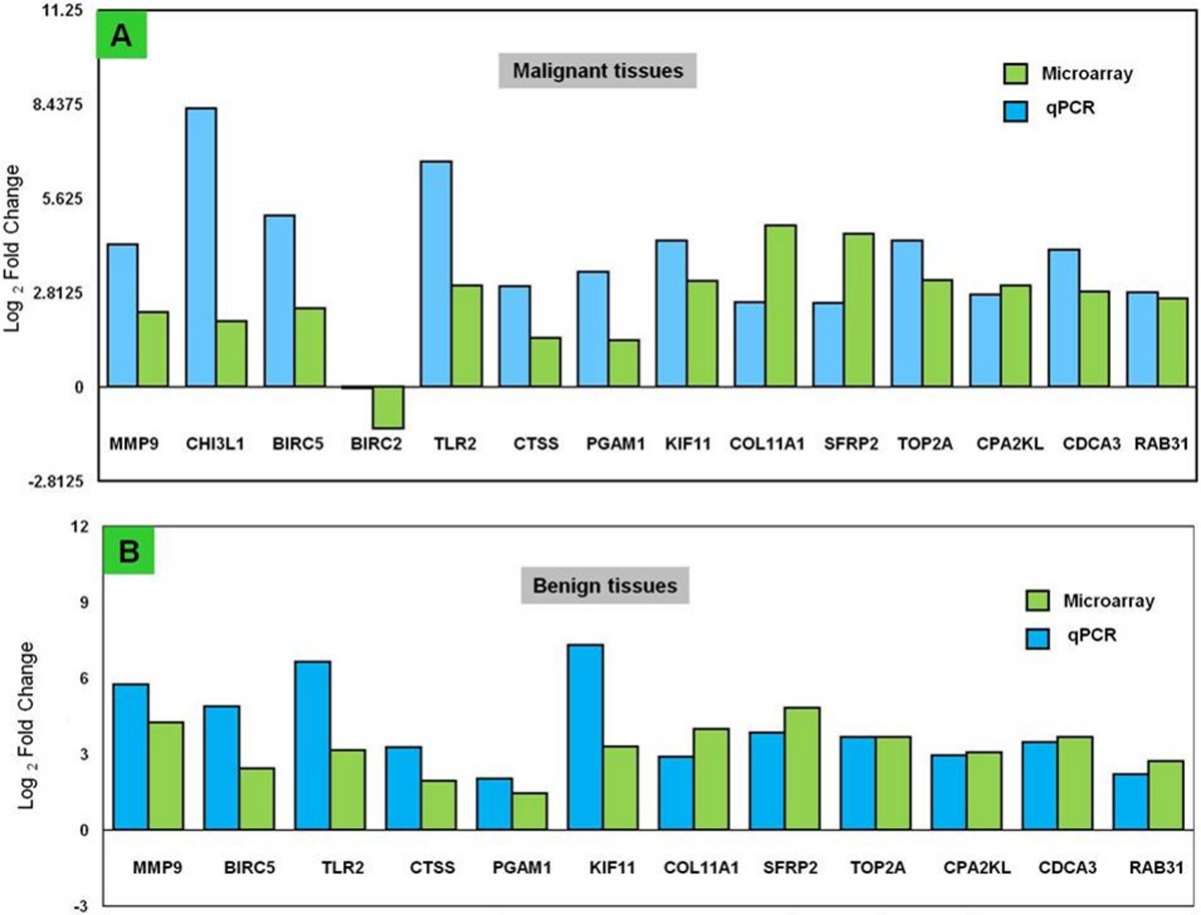
Fold change concordance of selected dysregulated genes by qPCR. The expression levels of the selected genes were compared between microarray and qPCR analysis. For microarray analysis, mean normalised signals from malignant and tissues were clubbed separately for comparison of gene expression levels. The expression levels represent log_2_ fold change values calculated from the normalised signal intensity values using healthy mammary tissues expression data as control. For qPCR analysis, gene expression in each sample was normalized against the expression of β-actin gene. The relative expression of each sample was calculated using the 2−ΔΔCT method with healthy group as calibrator and the log2fold change (log_2_FC) was plotted.

Microarray results were validated by carrying out qPCR analysis for selected genes viz., *MMP9*, *CHI3L1*, *BIRC5*, *BIRC2*, *TLR2*, *CTSS*, *PGAM*, *KIF11*, *COL11A1*, *SFRP2*, *TOP2A*, *CPA2KL*, *CDCA3*, *RAB31* in the same tissues used for microarray gene expression analysis. The genes selected for validation of microarray results included some of the top up-regulated genes, some randomly selected genes, as well as, some genes having potential role in CMT pathogenesis. The expression levels of the selected genes, in both microarray and qPCR technique were compared and results of both the studies revealed dysregulation of the target genes in a similar fashion (Fig 3).

### Differentially up-regulated proteins in CMTs

**Fig 4:**
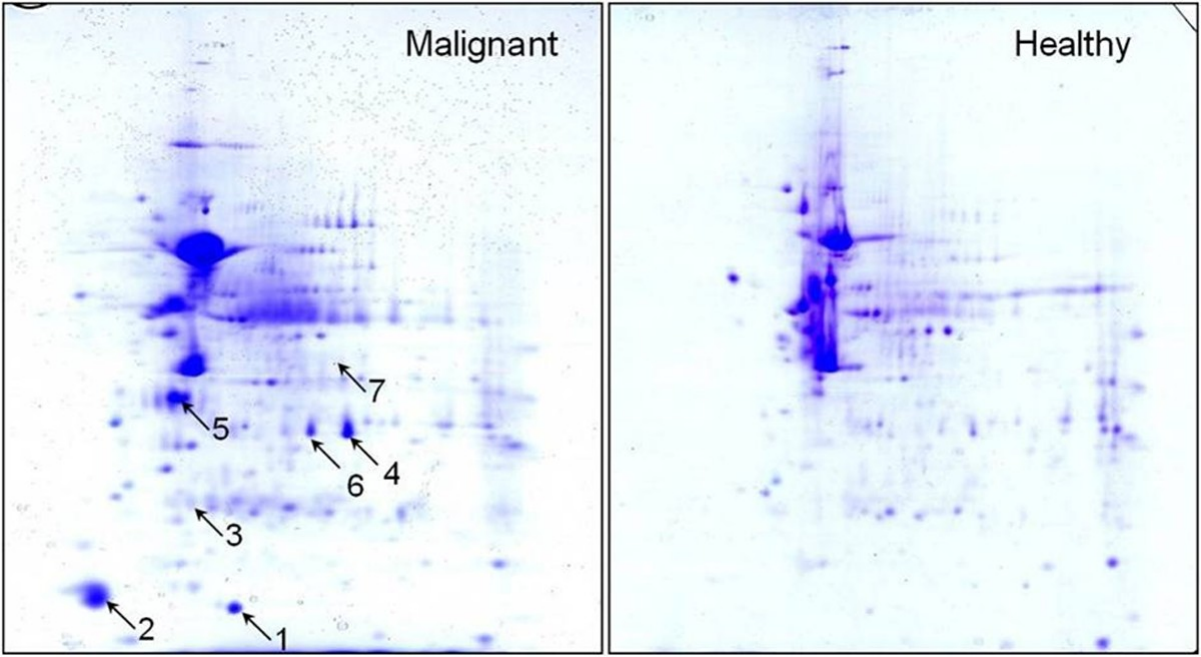
2D gel electrophoresis of malignant CMT versus healthy mammary gland tissues. Upon analysis of 2D gels of malignant versus healthy mammary tissue, 7 differentially expressed spots, (indicated by arrows 1–7) were identified in malignant tissue.

For identification of up-regulated proteins in malignant mammary tumours, total protein content from malignant and healthy mammary tissues was resolved separately using 2DGE. Upon analysis using ImageMaster program, 298 individual spots were identified in the 2-D gel from malignant tissue, whereas, 328 spots protein spots were identified in healthy mammary tissue. Out of these, 178 proteins spots matched in both cancer and healthy mammary tissues. After analysis of 2-D gels, we observed a total of 7 differentially expressed proteins (DEPs) having greater than three times higher expression level in malignant tissue as compared to healthy tissue (Fig 4). Upon MALDI-TOF PMF analysis, these proteins were confirmed as ANXA2, APOCII, CDK6, GATC, GDI2, GNAQ and MYH9. These 7 proteins were also found to be up-regulated in malignant tissues by microarray analysis, thus providing further validation of microarray data and confirming the overexpression of these genes at protein level in malignant canine mammary cancers.

### Relevant functions, pathways and biological networks in CMTs

Significant DEGs (*P ≤* 0.05) were used for functional annotation by Ingenuity Pathway Analysis (IPA) tool to explore the relevant biological pathways and functions of candidate genes involved in pathogenesis of CMTs. In IPA, the reference knowledge base was kept as *Homo sapiens* and *Mus musculus*, as most functional studies on cancer has been carried out in these organisms only and there is a severe lack of functional studies in CMTs. Each gene symbol was mapped to their corresponding identifiers in the Ingenuity knowledge base. Of the total 2336 genes uploaded into IPA, 2259 genes were mapped in the IPA knowledge base, whereas 77 genes remained unmapped. Networks were generated based on an algorithmically generated score based on connectivity between genes. A numerical value (Z-score) was used to rank networks according to how relevant they were to genes presented within the data set. Identified networks between genes are presented as a graph, indicating relationships between different genes in the data set. Genes are presented as nodes and relationship between two is indicated as a line.

### Canonical pathways deregulated in CMTs

**Fig 5:**
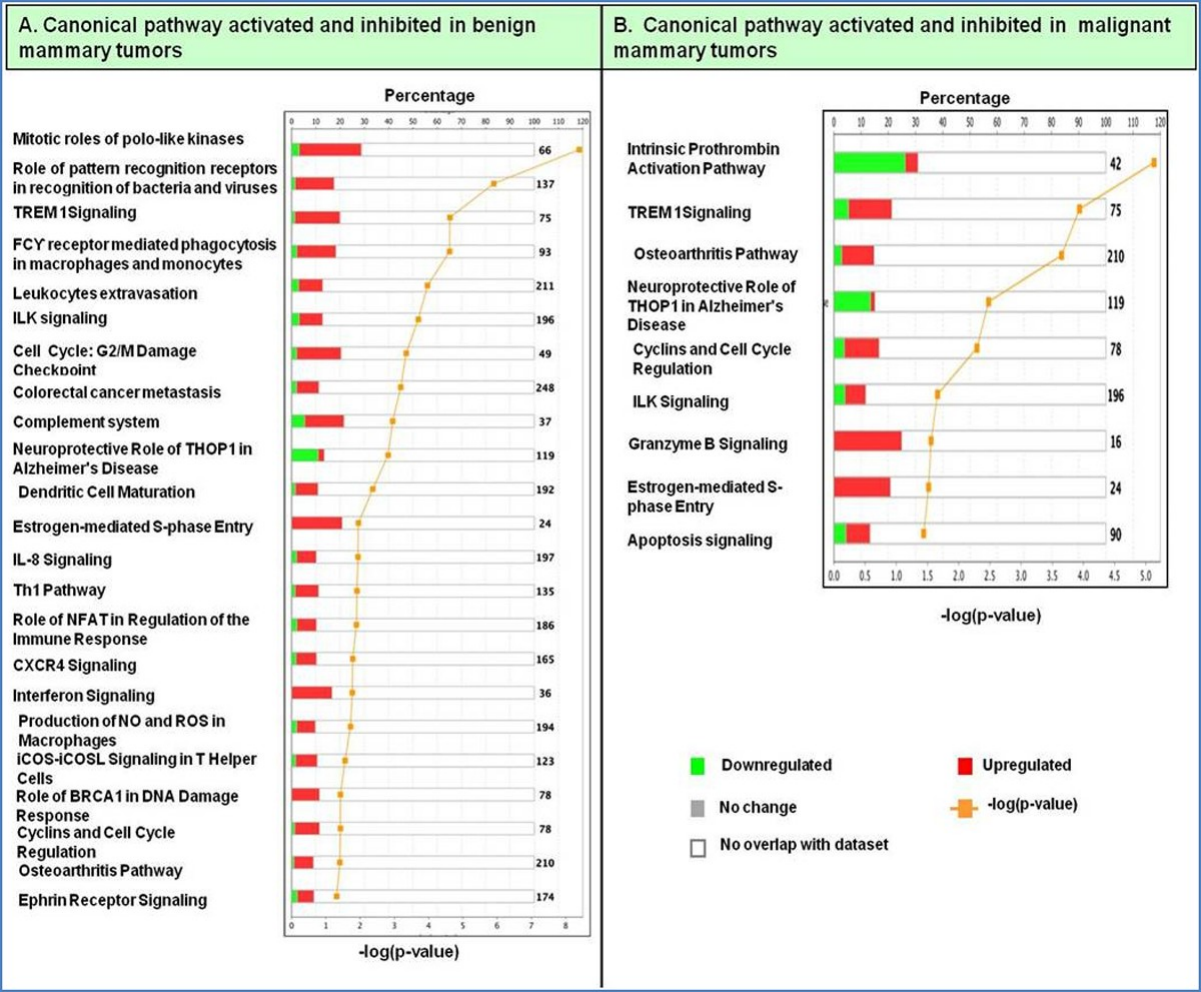
Top Canonical pathways identified using IPA in benign (A) and malignant (B) CMTs.

**Fig 6:**
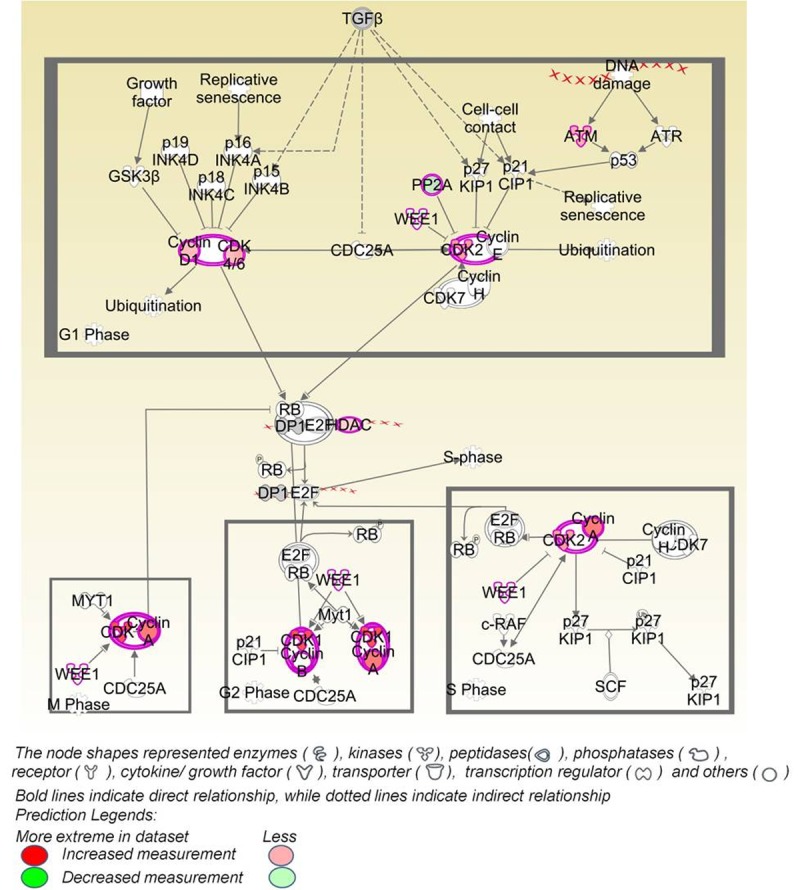
The top-ranked enriched canonical pathway identified in malignant CMTs using IPA: Cyclins and cell cycle regulation pathway.

**Fig 7:**
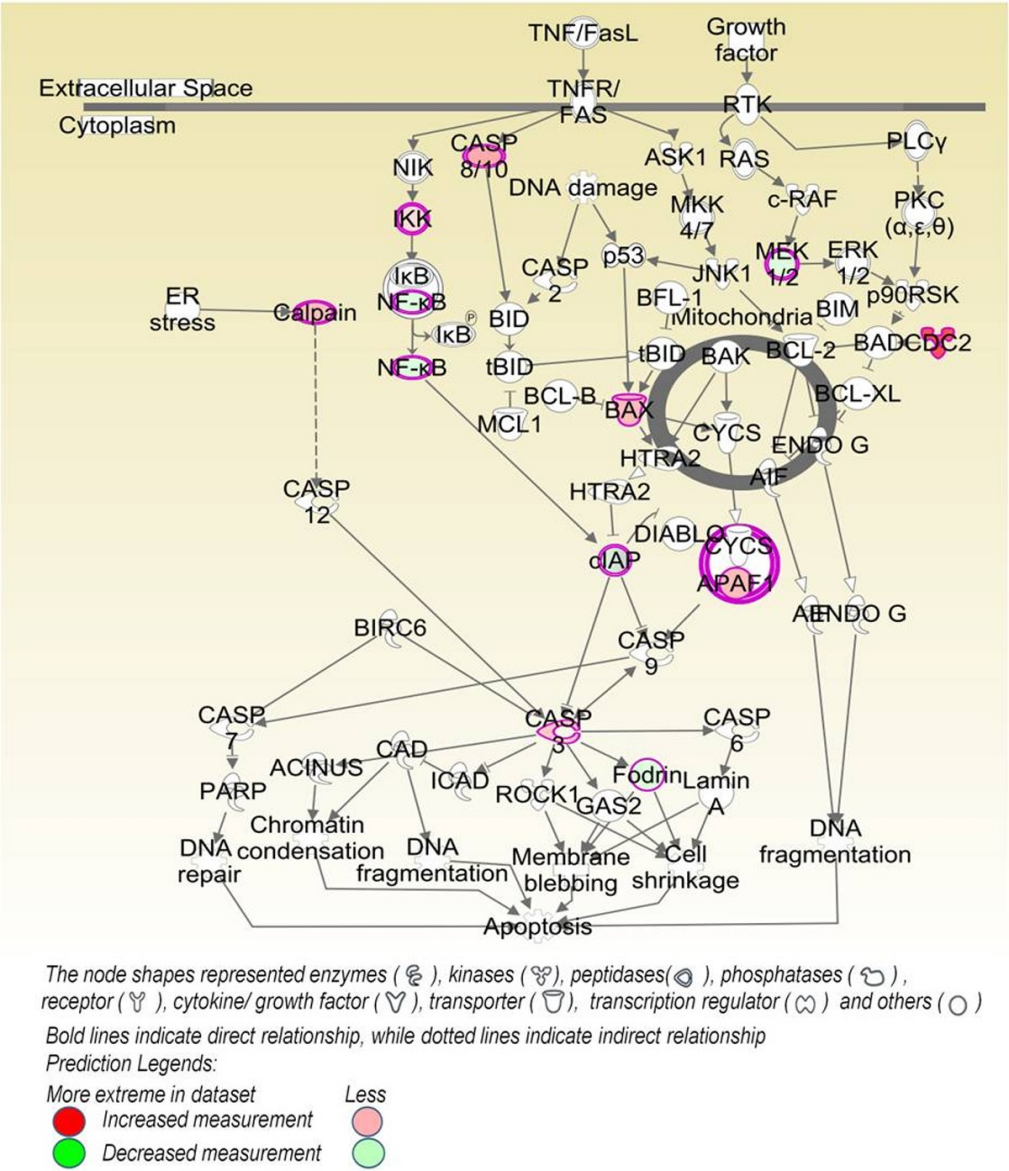
Apoptosis signalling pathway: The second-ranked enriched canonical pathway identified in malignant CMTs.

**Fig 8:**
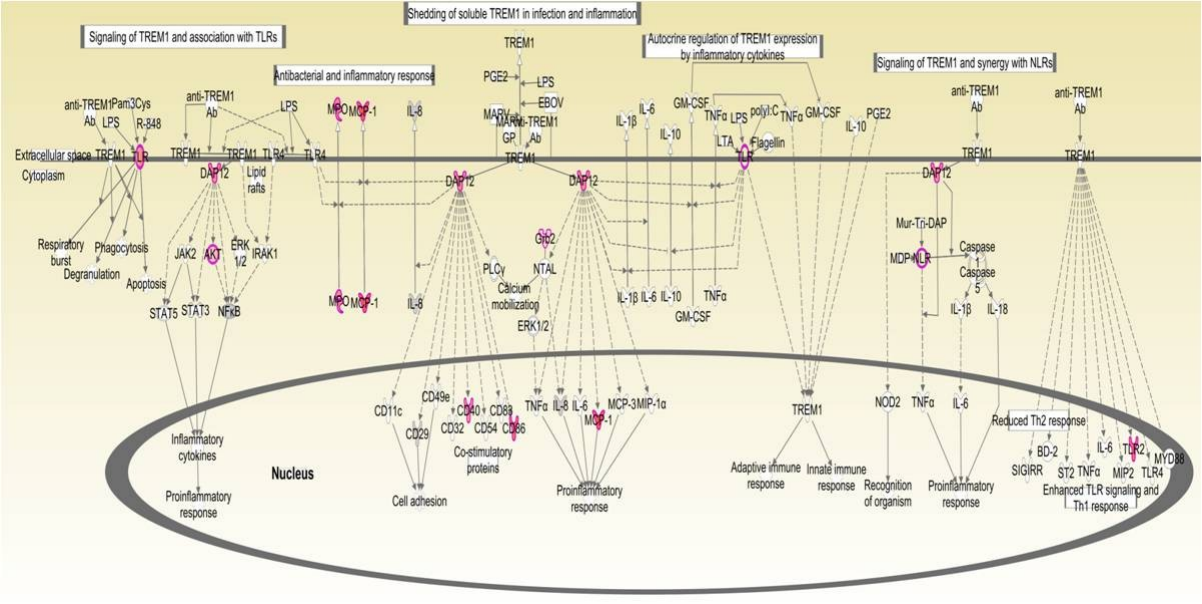
The top-ranked enriched canonical pathway identified in benign CMTs using IPA: The TREM1 signalling pathway.

**Fig 9:**
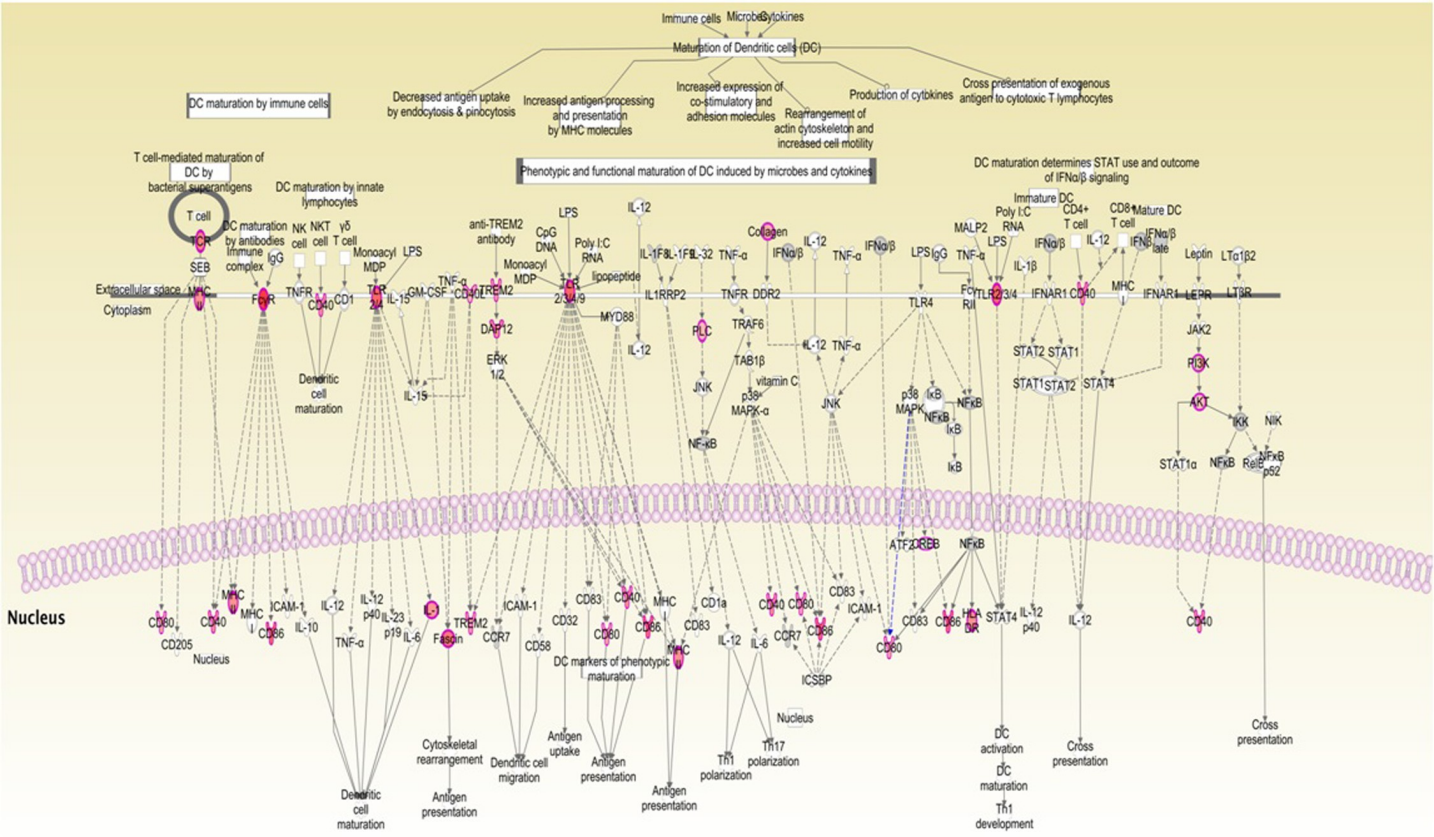
Dendritic cell maturation: Second enriched canonical pathway in benign CMTs, identified using IPA.

Upon investigating the interactions using the IPA software, 2259 functional pathway eligible genes were found. Canonical pathways, defined as the idealized or generalized pathways representing common properties of a particular signalling module or pathway, were identified by IPA. Top activated canonical pathways in malignant tumours, (z-score > 2) included cyclins and cell cycle regulation, apoptosis, and PPAR signalling pathway etc., and are shown in Table 1 and Fig 5B. Top inhibited pathways in malignant cancers included intrinsic prothrombin activation pathway and neuroprotective role of THOP1 in Alzheimer’s disease. For benign tumours, TREM1 signalling, dendritic cell maturation, Integrin linked kinase (ILK) signalling pathway etc., were amongst the top activated canonical pathways, while top inhibited pathways were related to cell cycle regulation, complement system and neuroprotective role of THOP1 in Alzheimer's disease (Fig 5A and Table 2). The functional relevance of the dysregulated genes in our dataset, related to the top activated pathways in malignant and benign CMTs is depicted in Figs 6–9.

**Table 1:** Canonical pathways with highest enrichment scores (z-score) in malignant CMTs and associated DEGs.

Ingenuity Canonical Pathways	Z- score	Differentially expressed genes
**TOP ACTIVATED PATHWAYS**		
Cyclins and Cell Cycle Regulation	2.887E+00	CCNB3,CDK4,ATM,CDK2,PPP2R2C,CCNB2,WEE1,CCND1,PPP2CB,CCNA2,CDK6,HDAC1,CDK1
PPARÎ±/RXRÎ± Activation	2.309E+00	CHUK,GNAS,ADCY3,LPL,INSR,IL1R2,INS,MAP2K1,NOTUM,PRKAR1A,NR0B2,RELA
Apoptosis Signalling	2.309E+00	BIRC2,CASP10,CHUK,APAF1,CASP3,BAX,MAP2K1,CAPNS1,CDK1,CASP8,SPTAN1,RELA
Estrogen-mediated S-phase Entry	2.236E+00	CDK4,CCND1,CDK2,CCNA2,CDK1
Osteoarthritis Pathway	2.191E+00	AGER,MMP9,FZD2,MMP12,IL1R2,RARRES2,FN1,SMAD7,ITGB1,GDF5,LRP1,CASP4,SPP1,TLR2,CASP10,CHUK,CTNNB1,H19,PRG4,SMAD9,CXCL8,RELA,PPARD,CREB5,HIF1A,CASP3,COL2A1,PTHLH,FOXO3,RUNX2,CASP8
ILK Signalling	2.132E+00	FERMT2,MYL4,MMP9,VCL,CTNNB1,ATM,MYL6B,ACTN1,RHOQ,PPP2R2C,PIK3CG,RELA,FN1,CREB5,MYH4,CCND1,ITGB1,HIF1A,PPP2CB,CASP3,RHOF,MYL7,PIK3C2G
Induction of Apoptosis by HIV1	2.121E+00	BIRC2,CXCR4,CHUK,APAF1,CASP3,BAX,CASP8,RELA
Cytotoxic T Lymphocyte-mediated Apoptosis of Target Cells	2.000E+00	APAF1,CASP3,FCER1G,CASP8
Tumouricidal Function of Hepatic Natural Killer Cells	2.000E+00	APAF1,CASP3,BAX,CASP8
Inflammasome pathway	2.000E+00	NLRP3,CTSB,CASP8,CXCL8
Granzyme B Signalling	2.000E+00	APAF1,LMNB1,CASP3,CASP8
TREM1 Signalling	2.000E+00	TLR1,MPO,NLRP9,CD40,CXCL8,RELA,TLR8,NLRP10,NLRP3,NLRP14,ITGB1,TYROBP,CCL2,TLR2,CD86,CIITA
**TOP INHIBITED PATHWAYS**		
MIF-mediated Glucocorticoid Regulation	-2.000E+00	PLA2G4E,PLA2G1B,PLA2G12B,RELA
p38 MAPK Signalling	-2.121E+00	RPS6KB2,PLA2G4E,CREB5,PLA2G1B,IL36B,PLA2G12B,IL1R2,EEF2K,IL1F10
Ceramide Signalling	-2.121E+00	ATM,PPP2CB,PPP2R2C,MAP2K1,PIK3C2G,PIK3CG,S1PR1,RELA
Huntington's Disease Signalling	-2.138E+00	APAF1,DNM1,UBE2S,ITPR1,GNG13,CASP4,HDAC1,BDNF,CAPNS1,PIK3C2G,RCOR1,CASP10,ATM,POLR2C,RPH3A,PIK3CG,IGF1R,REST,EGF,CREB5,DNM2,CASP3,BAX,CASP8,STX1A
Intrinsic Prothrombin Activation Pathway	-2.309E+00	KLK10,F10,F5,KLK14,KLK1,KNG1,FGB,COL2A1,KLK15,KLK9,F8,KLK8,F2
Neuroprotective Role of THOP1 in Alzheimer's Disease	-3.742E+00	SST,PRSS53,PRSS48,MMP9,CTRL,F7,KLK10,PRSS23,PRKAR1A,KLK1,KNG1,ECE1,CTRB2,PRSS2,KLK15,CFD,KLK8,PRSS36

**Table 2:** Canonical pathways with highest enrichment scores (z-score) in benign CMTs and associated DEGs.

Ingenuity Canonical Pathways	Z-Score	Differentially expressed genes
**TOP ACTIVATED PATHWAYS**		
TREM1 Signalling	3.36E+00	TLR7,AKT3,AKT1,TLR1,MPO,CD40,TLR8,NLRP3,NLRP14,TYROBP,CCL2,GRB2,TLR2,CD86,CIITA
Dendritic Cell Maturation	3.13E+00	AKT3,AKT1,FCGR1A,PLCB2,FCER1G,CD40,TREM2,PIK3CG,HLA-DRB1, CD40LG, ATF4, TYROBP, KLB, GRB2, CD80, COL11A2, TLR2, CD86, IL33, FSCN1, PIK3C2G
Role of Pattern Recognition Receptors in Recognition of Bacteria and Viruses	2.84E+00	EIF2AK2,OAS1,TLR7,TGFB1,TLR1,C1QB,OAS2,DDX58,IL13,C3AR1,C1QA,PIK3CG,C1QC,TLR8,PRKCD,SYK,C5AR1,NLRP3,OAS3,KLB,GRB2,TLR2,CLEC7A,PIK3C2G
ILK Signalling	2.71E+00	RHOH,AKT3,AKT1,MMP9,ITGB2,RHOQ,FN1,CFL1,ARHGEF6,MYH4,SNAI2,KLB,FBLIM1,GRB2,FLNC,PIK3C2G,CTNNB1,LEF1,ITGB8,ACTN1,CFL2,PIK3CG,ATF4,HIF1A,NACA
Role of NFAT in Regulation of the Immune Response	2.67E+00	GNA13,AKT3,AKT1,FCGR1A,PLCB2,CD79B,FCER1G,RCAN2,PIK3CG,XPO1,SYK,HLA-DRB1, KLB, GRB2, CD80, CSNK1G1, CD86, PIK3C2G, LYN
FcÎ³ Receptor-mediated Phagocytosis in Macrophages and Monocytes	2.67E+00	HCK,AKT3,AKT1,GPLD1,FCGR1A,NCF1,FGR,FYB,CBL,VAV1,ARPC1B,HMOX1,PIK3CG,PRKCD,SYK,PIK3C2G,LYN
G Beta Gamma Signalling	2.65E+00	PRKCD,GNA13,ARHGEF6,AKT3,AKT1,GRB2,PIK3CG
Colorectal Cancer Metastasis Signalling	2.65E+00	RHOH,AKT3,AKT1,TGFB1,MMP9,TLR1,ADCY6,FZD2,MMP12,RHOQ,TLR8,ADCY3,KLB,GRB2,TLR2,PIK3C2G,TLR7,CTNNB1,ADCY7,LEF1,BIRC5,WNT1,PIK3CG,MMP7,EGF,MMP3,BAX,MMP1
Leukocyte Extravasation Signalling	2.56E+00	CLDN10,RHOH,MMP9,MMP12,NCF1,ITGB2,WIPF1,CXCR4,KLB,GRB2,PIK3C2G,NCF4,CTNNB1,MSN,CLDN18,ACTN1,CLDN6,VAV1,EDIL3,PIK3CG,MMP7,PRKCD,MMP3,TIMP1,CYBB,MMP1,PECAM1
Th1 Pathway	2.50E+00	LGALS9,IFNGR2,ITGB2,CD40,VAV1,NOTCH2,PIK3CG,CCR5,HLA-DRB1,CD40LG,KLB,GRB2,CD80,CD86,PIK3C2G
Interferon Signalling	2.45E+00	OAS1,IFNGR2,ISG15,MX1,BAX,PSMB8
Production of Nitric Oxide and Reactive Oxygen Species in Macrophages	2.36E+00	NCF4,RHOH,AKT3,AKT1,IFNGR2,MPO,NCF1,RHOQ,PIK3CG,LYZ,PRKCD,APOB,MAP3K1,KLB,GRB2,SERPINA1,CYBB,TLR2,PIK3C2G
Mitotic Roles of Polo-Like Kinase	2.36E+00	CCNB3,TGFB1,PLK5,ESPL1,CDC20,CCNB2,KIF11,WEE1,CDC25C,ANAPC10,CDC7,PLK3,CDC16,PLK4,PLK2,PTTG1,PLK1,CDK1,CDC27
Cholecystokinin/Gastrin-mediated Signalling	2.33E+00	SST,RHOH,CREM,PRKCD,GNA13,PLCB2,GRB2,RHOQ,IL33
Cyclins and Cell Cycle Regulation	2.33E+00	CCNB3,CDK4,TGFB1,CDK2,CCNA2,CCNB2,TFDP1,WEE1,CDK1
Integrin Signalling	2.32E+00	RHOH,AKT3,AKT1,ITGB8,ITGB2,ACTN1,WIPF1,RHOQ,ARPC1B,PIK3CG,KLB,GRB2,ARF3,CAPNS1,PIK3C2G
Tec Kinase Signalling	2.31E+00	RHOH,HCK,GNA13,FGR,FCER1G,RHOQ,VAV1,PIK3CG,PRKCD,KLB,GRB2,PIK3C2G,LYN
HMGB1 Signalling	2.31E+00	RHOH,AKT3,AKT1,TGFB1,IFNGR2,CCL2,KLB,GRB2,IL13,RHOQ,PIK3C2G,PIK3CG
iCOS-iCOSL Signalling in T Helper Cells	2.31E+00	AKT3,AKT1,FCER1G,CD40,VAV1,PIK3CG,HLA-DRB1,CD40LG,KLB,GRB2,CD80,PTPRC,PIK3C2G
Role of BRCA1 in DNA Damage Response	2.24E+00	RFC3,RFC2,FANCA,HLTF,BRCA2,FANCM,RAD51,SMARCC1,PLK1
IL-8 Signalling	2.24E+00	RHOH,GNA13,AKT3,AKT1,MMP9,GPLD1,PLCB2,MPO,ITGB2,RHOQ,HMOX1,PIK3CG,EGF,PRKCD,KLB,FLT1,GRB2,BAX,CYBB,PIK3C2G
**TOP INHIBITED PATHWAYS**		
Complement System	2.24E+00	C1QC,C5AR1,C1QB,C4BPA,ITGB2,C6,C3AR1,C1QA
Cell Cycle: G2/M DNA Damage Checkpoint Regulation	-2.83E+00	TOP2A,CCNB3,AURKA,CKS2,CCNB2,YWHAH,WEE1,CDC25C,PLK1,CDK1
Neuroprotective Role of THOP1 in Alzheimer's Disease	-2.89E+00	SST,PRSS53,TAC1,MMP9,CTRL,F7,PREP,KLK1,KNG1,ECE1,CTRB2,PRSS2,KLK15,PRSS12,KLK8,PRSS36

**Fig 10:**
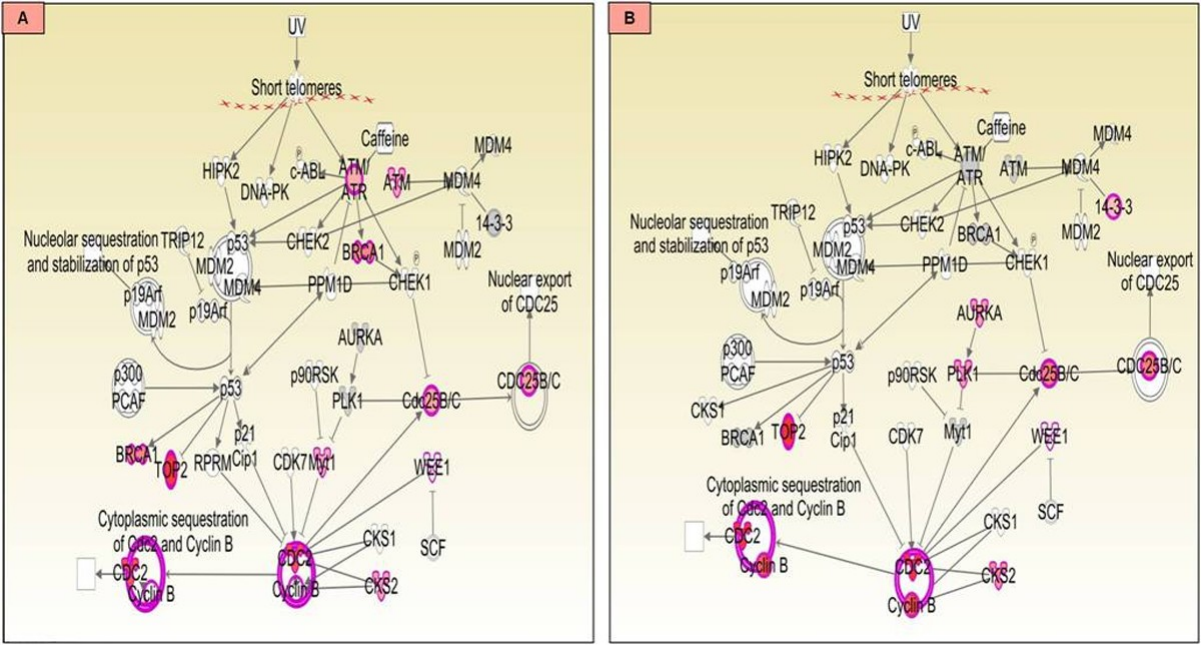
Canonical pathway–G2/M DNA damage checkpoint in (A) malignant and (B) benign CMTs.

The DNA damage checkpoint, occurs at the end of G2 and prevents DNA damage that might have occurred during replication. Any defects in this pathway results in genome instability and leads to carcinogenesis. Upon comparison of this pathway in malignant and benign tumours, it was seen that, in malignant tissues ATM/ATR kinases having role in controlling BRCA1 phosphorylation *in vivo* are involved in regulation of G2/M DNA damage checkpoint, while in benign tissues, activation of alternative AURKA–PLK1 pathway may play that role (Fig 10). Other important canonical pathways related to cancer such polo like kinase related pathways, cyclins and cell cycle regulation and Wnt/β-catenin signalling pathways were also compared between malignant and benign CMTs (S1–S5 Figs), highlighting the differential involvement of dysregulated genes contributing to malignant or benign nature.

### Upstream regulators identified in CMTs

**Fig 11:**
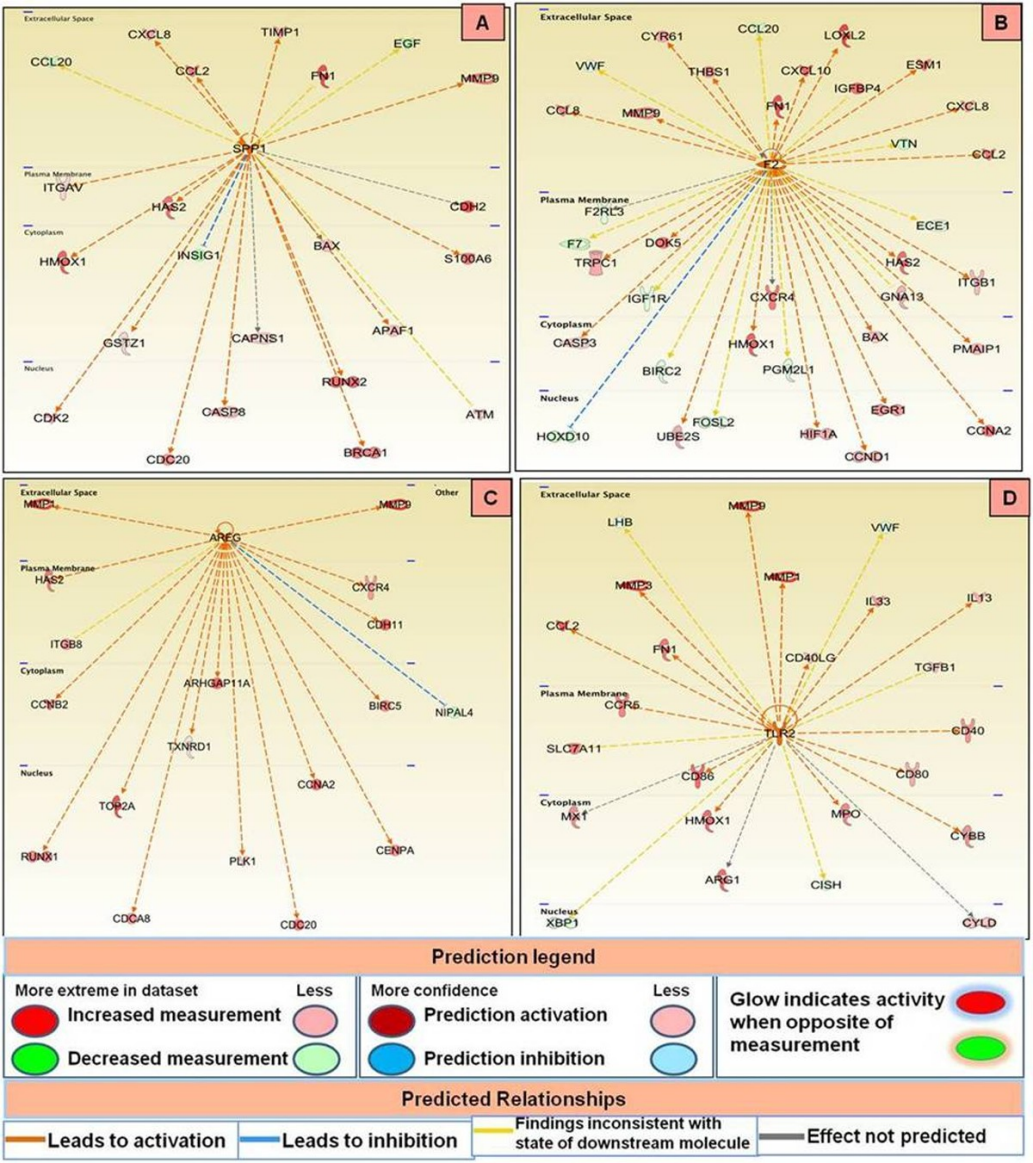
Various genes affected by top activated upstream regulators in malignant (A and B) and benign (C and D) CMTs.

**Fig 12:**
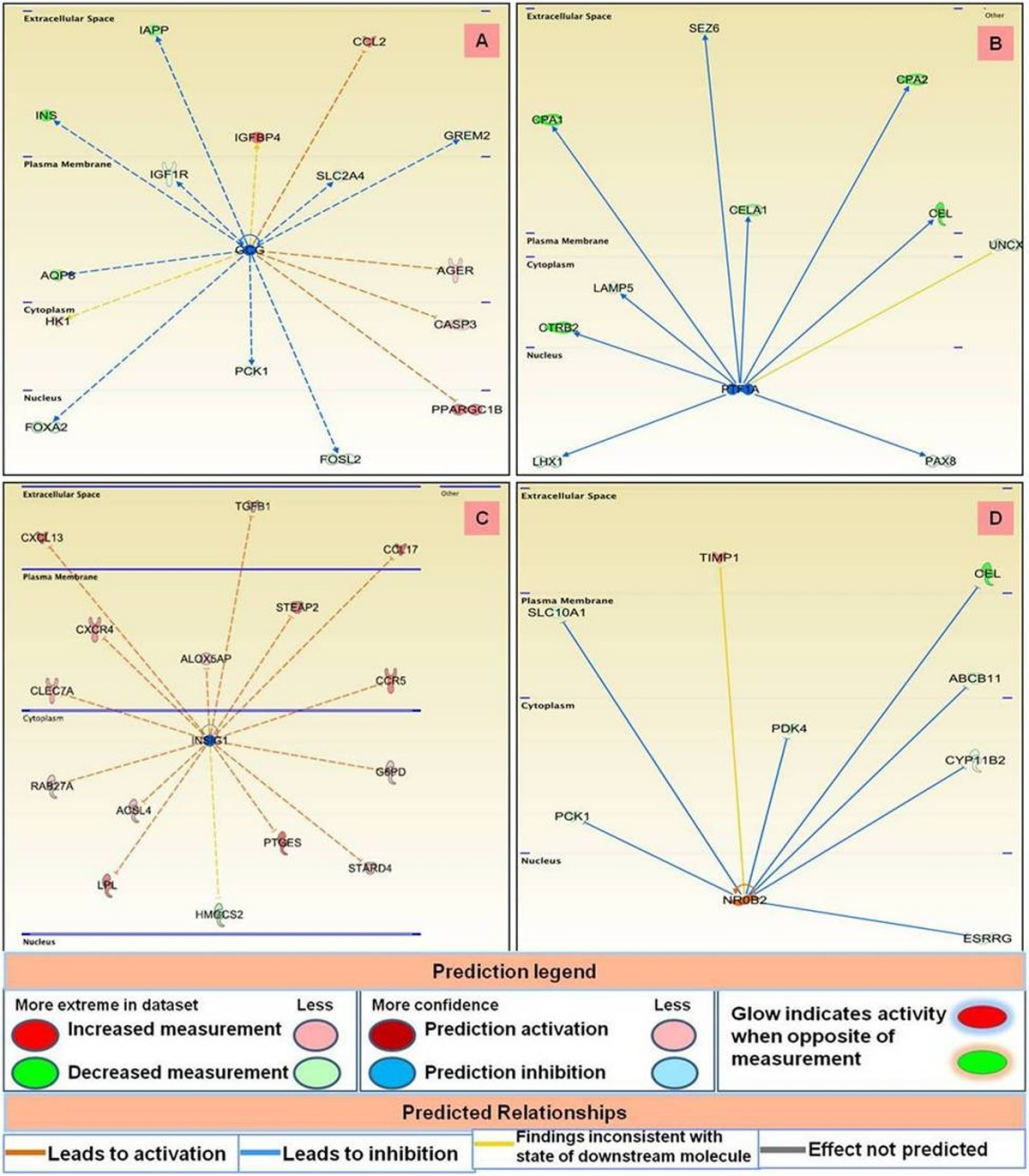
Various genes affected by top inhibited upstream regulators in malignant (A and B) and benign (C and D) CMTs.

Upstream regulators have been defined as genes that direct the expression of numerous other genes such that dysregulation of an upstream regulator affects the expression level of downstream gene it affects. In malignant tumours, 24 upstream regulators were identified which were mainly enzymes, transcription regulators, G protein coupled receptors, kinases, etc. (Table 3). The prediction of activation state was based upon the global direction of changes of the modulated genes. The activation Z-score, indicating whether the observed gene responses to upstream regulators agree with expected changes derived from the literature that accrued in the Ingenuity Knowledge Base, was used to predict the activation state. Pathway analysis revealed that secreted phosphoprotein (SPP1) gene was the top activated upstream regulator among malignant tissues and affected expression of various genes including expression levels of breast cancer type 1 susceptibility protein (BRCA1) and cell-division cycle protein 20 (CDC20). Various genes affected by SPP1 are displayed in Fig 11A. Analysis of the malignant data set also revealed F2 (coagulation factor II, Thrombin) as another top activated upstream regulator, which was connected with overexpression of chemokines CCL2, CXCL8, CXCL10, CCL8 and their ligands CXCR4, F2RL2, ITGB1 (Fig 11B). Other upstream regulators like GCG and PTF1A were found to be downregulated and inhibited the expression of various associated genes as depicted in Fig 12A and 12B. In benign tumours, 25 upstream regulators were identified. Of these 22 were activated, and 3 were inhibited based on Z-score cut-off (Table 4). The genes affected by the top activated upstream regulators in benign tissues i.e., AREG and TLR2 are displayed in Fig 11C and 11D. Analysis also revealed INSIG1 and NROB2 as the top inhibited upstream regulators among the benign cancers.

**Table 3:** Upstream regulators in malignant CMTs.

Upstream Regulator	Expression Log Ratio	Molecule Type	Predicted Activation State	Z-score
FN1	2.828	Enzyme	Activated	2.277
SPP1	2.384	Cytokine	Activated	2.978
CXCR4	2.115	G-protein coupled receptor	Activated	2.556
PPARGC1B	1.867	Transcription regulator	Inhibited	-2.395
EGR1	1.592	Transcription regulator	Activated	2.174
TYROBP	1.558	Transmembrane receptor	Activated	2.2
CYR61	1.444	Other	Activated	2.812
EIF2AK2	1.341	Kinase	Activated	2.179
C5AR1	1.24	G-protein coupled receptor	Activated	2.76
MTPN	1.222	Transcription regulator	Activated	3.081
CCND1	1.059	Transcription regulator	Activated	3.681
SRC	0.993	Kinase	Activated	2.13
CASP3	0.889	Peptidase	Activated	2.356
CHUK	0.569	Kinase	Activated	2.649
LHX1	-1.042	Transcription regulator	Inhibited	-2.111
NUPR1	-1.423	Transcription regulator	Inhibited	-4.866
ALDH1A2	-1.591	Enzyme	Inhibited	-2.646
IFRD1	-1.88	Other	Inhibited	-2.213
HNF1B	-2.604	Transcription regulator	Inhibited	-2.331
NR5A2	-2.698	Ligand-dependent nuclear receptor	Inhibited	-2.976
EGF	-3.865	Growth factor	Activated	3.333
PTF1A	-4.347	Transcription regulator	Inhibited	-2.562
GCG	-4.56	Other	Inhibited	-2.842
F2	-7.153	Peptidase	Activated	2.501

**Table 4:** Upstream regulators in benign CMTs.

Upstream Regulator	Expression Log Ratio	Molecule Type	Predicted Activation State	Z-score
AREG	5.72	Growth factor	Activated	3.882
TLR2	3.158	Transmembrane receptor	Activated	2.392
PTGES	3.026	Enzyme	Activated	2.193
FN1	2.834	Enzyme	Activated	2.664
HGF	2.675	Growth factor	Activated	3.886
NCF1	2.566	Enzyme	Activated	2.219
C5AR1	2.336	G-protein coupled receptor	Activated	3.251
TYROBP	2.272	Transmembrane receptor	Activated	2.224
CD40	2.233	Transmembrane receptor	Activated	2.081
CTLA4	2.201	Transmembrane receptor	Inhibited	-2.326
EIF2AK2	2.011	Kinase	Activated	2.575
REL	1.868	Transcription regulator	Activated	2.663
ITGB2	1.801	Transmembrane receptor	Activated	2.425
CD40LG	1.484	Cytokine	Activated	3.583
C3AR1	1.426	G-protein coupled receptor	Activated	2.395
TLR7	1.425	Transmembrane receptor	Activated	3.851
TGFB1	1.402	Growth factor	Activated	5.165
HIF1A	0.888	Transcription regulator	Activated	2.939
AKT1	0.809	Kinase	Activated	2.41
MAP3K1	-0.841	Kinase	Activated	2.905
NUPR1	-2.063	Transcription regulator	Inhibited	-5.986
INSIG1	-3.015	Other	Inhibited	-3.357
EGF	-3.154	Growth factor	Activated	4.898
NR0B2	-4.416	Ligand-dependent nuclear receptor	Activated	2.16
F2	-7.006	Peptidase	Activated	3.085

Common regulators that were affecting the dysregulation of genes in malignant tumours had a consistency score ranging from 9.9 to -13.584. Further, 47 regulators have a combined consistency score of 9.9 which affected a total of 142 genes in our data set, followed by a group of 27 regulators which affected 173 genes having an overall consistency score of 9.7. These two groups of regulators affected diseases related to female genital neoplasm, hematologic cancer, lymphocytic neoplasm, lymphoid cancer, tumourigenesis of reproductive tract and hematologic cancer, lymphocytic neoplasm, lymphoid cancer. Similarly, common regulators were also identified for benign tumours with consistency score ranging from 3.7 to -12.2. The regulators with the highest score were related to cell movement, invasion of cells and cell movement of tumour cell lines.

### Functional relevance of activated pathways and networks in CMTs

For knowing the functional relevance of activated pathways and networks, we investigated the disease and bio-functions related to the top activated, as well as, inhibited pathways using IPA tool. The top most affected diseases with respect to genes in our malignant data set were cancer, organismal injury and abnormalities, etc. The activated bio-functions in malignant tissues were attributed to cell to cell signalling, cellular movement, inflammatory response, immune cell trafficking, DNA replication, recombination, and repair. Genes that were seen to be down-regulated in our analysis were related to bio-functions like exocytosis, secretory pathways, bacterial infections, female genital neoplasms etc. (Table 5). Among the benign tumours, top most activated bio-functions were related to immune response of leukocytes, immune response of cells, cell engulfment and cell survival. The top inhibited bio-functions were related to organismal death, morbidity or mortality (Table 6).

**Table 5:** Diseases and bio-functions associated with malignant CMTs and their association with the top signalling pathways.

Signalling Pathways	Diseases or Functions Annotation	p-Value	Predicted Activation State	Activation z-score
Cell-To-Cell Signalling and Interaction, Cellular Movement, Hematological System Development and Function, Immune Cell Trafficking	recruitment of antigen presenting cells	1.25E-05	Increased	3.494
Cell-To-Cell Signalling and Interaction, Cellular Movement, Hematological System Development and Function, Immune Cell Trafficking, Inflammatory Response	recruitment of macrophages	7.09E-06	Increased	3.397
Cellular Function and Maintenance	endocytosis	2.18E-05	Increased	3.339
DNA Replication, Recombination, and Repair	metabolism of DNA	6.81E-07	Increased	3.337
Cell-To-Cell Signalling and Interaction, Hematological System Development and Function	activation of myeloid cells	9.77E-06	Increased	3.069
Cellular Movement	cell movement of myeloid cells	6.51E-08	Increased	2.998
Inflammatory Response, Organismal Injury and Abnormalities	inflammation of organ	1.96E-09	Increased	2.939
Cell-To-Cell Signalling and Interaction, Hematological System Development and Function, Immune Cell Trafficking, Inflammatory Response	activation of phagocytes	3.06E-07	Increased	2.899
Cell-To-Cell Signalling and Interaction, Hematological System Development and Function, Immune Cell Trafficking, Inflammatory Response	activation of leukocytes	1.40E-09	Increased	2.233
Cardiovascular System Development and Function, Embryonic Development, Organ Development, Organismal Development, Tissue Development	cardiogenesis	1.50E-05	Increased	2.013
Cancer, Hematological Disease, Organismal Injury and Abnormalities	hematologic cancer	2.06E-07	Decreased	-2.012
Cellular Function and Maintenance, Molecular Transport	exocytosis	1.26E-05	Decreased	-2.012
Cellular Function and Maintenance, Molecular Transport	secretory pathway	1.39E-05	Decreased	-2.012
Infectious Diseases	Bacterial Infections	2.24E-06	Decreased	-2.052
Cancer, Organismal Injury and Abnormalities, Reproductive System Disease	female genital neoplasm	1.35E-15	Decreased	-2.056
Cancer, Organismal Injury and Abnormalities, Reproductive System Disease	tumourigenesis of reproductive tract	4.26E-15	Decreased	-2.056
Endocrine System Disorders, Organismal Injury and Abnormalities, Reproductive System Disease	ovarian lesion	1.48E-10	Decreased	-2.092
Hematological Disease, Immunological Disease	lymphoproliferative disorder	5.38E-07	Decreased	-2.144
Cancer, Hematological Disease, Organismal Injury and Abnormalities	lymphoid cancer	1.44E-06	Decreased	-2.24
Cancer, Hematological Disease, Organismal Injury and Abnormalities	lymphocytic neoplasm	2.30E-07	Decreased	-2.33

**Table 6:** Diseases and bio-functions associated with benign CMTs and their association with the top signalling pathways.

Categories	Diseases or Functions Annotation	p-Value	Predicted Activation State	Activation z-score
Cell-To-Cell Signalling and Interaction, Inflammatory Response	immune response of leukocytes	3.35E-10	Increased	5.376
Inflammatory Response	immune response of cells	1.07E-14	Increased	5.343
Cellular Function and Maintenance	engulfment of cells	6.72E-09	Increased	5.188
Cell Death and Survival	cell survival	2.18E-16	Increased	5.161
Cellular Function and Maintenance, Inflammatory Response	phagocytosis	3.44E-08	Increased	5.145
Cell-To-Cell Signalling and Interaction, Cellular Function and Maintenance, Inflammatory Response	phagocytosis of cells	2.60E-07	Increased	5.1
Cellular Function and Maintenance	endocytosis by eukaryotic cells	7.52E-07	Increased	5.044
Cell-To-Cell Signalling and Interaction, Inflammatory Response	response of phagocytes	7.89E-08	Increased	5.025
Cellular Movement, Hematological System Development and Function, Immune Cell Trafficking, Inflammatory Response	cell movement of phagocytes	1.55E-17	Increased	4.977
Cancer, Endocrine System Disorders, Organismal Injury and Abnormalities, Reproductive System Disease	Ovarian Cancer and Tumours	2.65E-09	Decreased	-2.186
Cancer, Gastrointestinal Disease, Organismal Injury and Abnormalities	colon cancer	2.48E-09	Decreased	-2.2
Cancer, Endocrine System Disorders, Organismal Injury and Abnormalities, Reproductive System Disease	gonadal tumour	8.87E-09	Decreased	-2.4
Developmental Disorder, Neurological Disease	congenital anomaly of central nervous system	6.50E-07	Decreased	-2.522
Cancer, Gastrointestinal Disease, Organismal Injury and Abnormalities	large intestine neoplasm	2.30E-24	Decreased	-2.612
Cancer, Gastrointestinal Disease, Organismal Injury and Abnormalities	colorectal neoplasia	3.63E-12	Decreased	-2.612
Infectious Diseases	infection of mammalia	1.10E-08	Decreased	-2.734
Cell Death and Survival	cell death of cervical cancer cell lines	1.00E-07	Decreased	-2.789
Cancer, Gastrointestinal Disease, Organismal Injury and Abnormalities	colon tumour	3.45E-09	Decreased	-2.957
Organismal Survival	morbidity or mortality	5.44E-24	Decreased	-5.407
Organismal Survival	organismal death	4.24E-24	Decreased	-5.493

**Fig 13:**
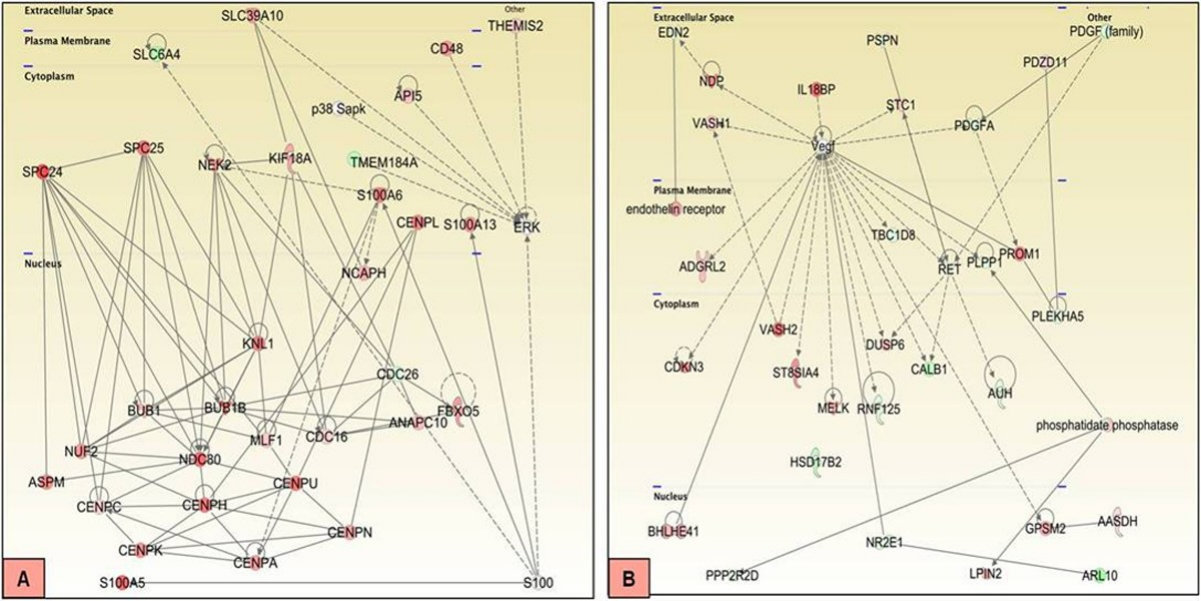
**Top scoring networks in malignant (A) and benign (B) CMTs.** Top scoring network in malignant CMTs involved a central node of BUB1B, while top network amongst benign tumours involved VEGF central hub.

To further understand the inter-relationship between genes that were dysregulated in malignant and benign tumours, networks were identified to present how the genes interact and coordinate their roles in a specific pathway. In malignant tumours, 25 networks were identified that were related to cell cycle, cellular assembly and organization, DNA replication, recombination, and repair. The network with a maximum score was related to cell cycle, cellular assembly and organization, DNA replication, recombination, and repair. Other top networks were related to developmental and hereditary disorders, organismal injury, abnormalities, post-translational modification, DNA replication, recombination and repair, cell cycle etc. Networks presenting interrelationship of genes dysregulated in benign tumours were 25 in total with a maximum score for the network showing genes related to cellular assembly and organization, cell cycle, DNA replication, recombination, and repair. Other notable identified networks were related to cancer, organismal injury, abnormalities and embryonic development, cell injury, DNA replication, cell signalling and cell cycle among others. It was observed that the top scoring network amongst the malignant mammary cancers have a central node of mitotic checkpoint serine/threonine kinase (BUB1B), while top network in the benign tumours have a central node of vascular endothelial growth factor (VEGF), as shown in Fig 13.

## Discussion

Mammary tumour is an important neoplastic disease of dogs, besides its role as a model for human breast cancer studies. Our study has revealed new biomarkers, networks and pathways dysregulated in canine mammary tumours. Previous work on expression profiling of canine mammary tumour has suggested that separate pathological and molecular characterization can complement one another [8]. Previous transcriptomic studies of canine mammary tumours (CMTs) have shown that transcriptomic signatures overlap with human breast cancer profiles and dogs are reliable models for oncogenic and pharmacogenomic studies [9].

### Clinical relevance of top up-regulated genes in cancer pathogenesis

The top up-regulated genes among malignant and benign tumours in our study were found to have a significant role in cancer progression and tumour invasiveness, as evidenced by human cancer studies. Top five up-regulated genes in malignant tumours were *COL11A1*, *SFRP2*, *LCN2*, *COL2A1* and *H19*. *COL11A1* gene encodes one of the two alpha chains of type XI collagen, a minor fibrillar collagen which has been implicated in tumour progression in humans. Overexpression of *COL11A1* has been reported in mesenchymal derived tumours [10–11]. Wu *et al*., reported that *COL11A1* may promote tumour aggressiveness via the TGF-β1 and MMP3 axis in ovarian cancers [12]. Halsted *et al*., reported expression of *COL11A1* in epithelial cells, stroma, and vessels of normal and cancerous breast tissue [13]. Overexpression of COL11A1 has recently been reported in canine melanomas [14]

Secreted frizzled-related proteins (SFRPs) constitute a family of 5 members (SFRP 1–5) that modulate Wnt signalling. Overexpression of secreted frizzled related protein 2 (SFRP2) in CMTs has been shown to induce cancerous transformation in normal mammary epithelial cells [15]. The anti-apoptotic function of SFRP2 is mediated through activation of NF-κB or Janus kinase (JNK) suppression [16]. Further *in vitro*, as well as, *in vivo* oncogenic potential of SFRP2 have been demonstrated in renal cancer. Recently, SFRP2 was shown to be related to poor prognosis along with genes associated with epithelial-to-mesenchymal transition [17]. Overexpression of SFRP2 was seen in both malignant and benign CMTs in this study. Lipocalin (LCN2), another top up-regulated gene among malignant tumours in our data set, has been shown to be associated with oestrogen receptor (ER)-negative breast tumours in humans [18]. The gene is significantly increased in the luminal epithelial cells compared with myoepithelial cells, an important finding because the majority of breast carcinomas are thought to arise from the luminal epithelial cells [19–20]. LCN2 promotes breast and prostate cancer progression by inducing epithelial to mesenchymal transition (EMT) through the ERα/Slug axis [21–22]. Structural similarity between human and dog LCN2 has been studied recently [23]. LCN2 has been shown to be associated with tumour invasiveness of human cervical cancer also [24]. Ganpathi *et al*., revealed that high expression of collagen type II, alpha 1 (*COL2A1*) was associated with delayed time to recurrence in high grade serious ovarian cancers [25]. *COL2A1* gene also undergoes somatic alterations in chondrosarcoma and enchondroma cases [26]. H19 is a gene for a long noncoding RNA, having a role in the negative regulation of body weight and cell proliferation [27]. Dugimont *et al*., reported expression of H19 gene in both epithelial and stromal components of human invasive adenocarcinomas [28]. H19 is associated with enhancer of EZH2, and this association results in Wnt/β-catenin activation and subsequent down-regulation of E-cadherin. H19 has been reported to enhance bladder cancer metastasis by associating with EZH2 and inhibiting E-cad expression [29]. Biological and clinical relevance of H19 in colorectal cancer patients has been reported recently [30].

The most up-regulated genes among benign mammary tumours in our experiment were MMP3, MMP1, AREG, PTHLH and SFRP2. Matrix metalloproteinases (MMPs) play role in cancer progression by degrading extracellular matrix and basement membrane and are the main proteolytic enzymes involved in cancer invasion and metastasis [31]. MMP3 and MMP1 have a synergistic effect on breast cancer carcinogenesis [32]. MMP1 is the most widely expressed collagenase and plays role in degradation of collagen I, II and III. MMP3 has a high proteolytic efficiency and activates a number of proMMPs [33–34]. Overexpression of MMPs has been reported widely in canine mammary tumours [9, 35].

Amphiregulin (AREG) is one of the many ligands for epithelial growth factor receptor [36]. It plays a central role in mammary gland development and branching morphogenesis in organs and is expressed both in healthy and cancerous tissues [37–40]. The oncogenic potential of AREG and its role in tissue invasion & metastasis, angiogenesis, resistance to apoptosis etc., has been reported in human epithelial malignancies, such as lung, breast, colorectal, ovary and prostate carcinomas, as well as, in some haematological and mesenchymal cancers [40–45]. Furthermore, AREG also contributes to therapeutic resistance to several cancer treatments [46].

Parathyroid hormone like hormone (PTHLH) has previously been reported to be produced by tumour cells in the bone microenvironment and is implicated in osteoclastic activity and bone metastasis [47]. Ghoussaini *et al*., combined several datasets for a genome wide analysis and identified PTHLH as loci for susceptibility for breast cancer [48]. Researchers suggest that PTHLP powerfully promotes tumour formation in breast cancer [49].

### Impact analysis of major upstream regulators reveals genes involved in tumour angiogenesis and cancer metastasis

In this study, we found numerous dysregulated upstream regulators in both malignant and benign tumours of mammary gland, which affected expression patterns of various other genes related with tumourigenesis. The top upstream regulators in malignant mammary tumours were secreted phosphoprotein (SPP1) and coagulation factor II (F2). Pathway analysis revealed that SPP1 overexpression affected the expression levels of BRCA1 and CDC20, which have been reported to be associated with several cancers types [50–51]. F2 (thrombin) in our study was found to be dysregulated and its expression has been connected to overexpression of various chemokines and their ligands having role in tumour angiogenesis and cancer metastasis [52–54]. Nierodzik and Karpatkin have provided ample evidence to support that thrombin activates tumour cell adhesion to platelets, endothelial cells, and subendothelial matrix proteins. Thrombin also enhances tumour cell growth, metastasis and angiogenesis, apart from its role in preservation of dormant tumour cells in individuals, preventing host eradication. Therefore, it is proposed that tumour malignancy may be regulated by a procoagulant/anticoagulant axis [55].

Top upstream regulator identified for benign mammary tumour was AREG. DNA damage signals caused by radiation and chemo are transmitted by master regulators like NF-kB to generate a powerful, conserved and senescence associated secretory phenotype, and its downstream effectors comprise a large spectrum of extracellular proteins including AREG, SFRP2, HGF, IL8, MMPs. Together these proteins give rise to a pro-angiogenic and pro-inflammatory micro-environmental niche that promotes malignant phenotype [56–57]. Furthermore, AREG affected downstream molecules like BIRC5, CCNA2, CCNB2, TOP2A, MMP9, MMP1, CXCR4, have roles in development and progression of various types of cancers [32, 57–61].

Important upstream regulators in our study, unique to benign tumours were AREG, TLR2, TGF1B, HGF, MAP3K1. TLR2 has been reported in intestinal and breast epithelia oncogenesis. Scheeren *et al*., reported that inhibition of TLR2 or its co-receptor CD14, or its downstream targets MYD88 and IRAK1 inhibits growth of human breast cancers *in vitro* and *in vivo* [62]. TLR2 agonists fed to neu transgenic mice significantly inhibits breast cancer growth [63] and leads to inhibition of immune responses by production of IL-10 and regulatory T-cells [64]. Thus, TLR2 stimulation on immune cells may also have opposing immune effects as in the case of PSA and PSK [65].

### Role of top activated pathways and networks in cancer pathogenesis

The top activated pathways in malignant tumours revealed a unique cancer landscape wherein induction of certain pathways involved targets associated with cell cycle regulation, cellular proliferation, apoptotic pathways, cellular stress and injury (e.g. pathways of cell cycle regulation, oestrogen mediated S phase entry, granzyme B signalling and apoptosis signalling). The data suggests genes involved in cell cycle regulation, apoptosis and cell signalling as major events in the study.

The top scoring network in canine mammary tumour was found to have BUB1B as central node in this study. BUB1B is a protein kinase involved in metaphase to anaphase transition checkpoint [66–67]. Aneuploidy and chromosomal instability (CIN) in human cancers can be attributed to alterations in this checkpoint [68]. BUB1B was seen to be linked with NDC80, an essential protein of kinetochore-associated complex required for chromosome segregation and spindle checkpoint activity [69]. NDC80 complex exists as part of a larger super complex called KMN network previously reported to be overexpressed in CMTs [9]. NDC80 has also been shown to depend on KNL-1 and the CENP/H/I/K complex for kinetochore recruitment [70–72]. A subnetwork of centromeric proteins CENP- H/C/U/K/A/N was seen within the network. Overexpression of CENP-H is reported in the development and pathogenesis of human breast, colorectal, oesophageal and oral squamous cell cancers [73–76].

Among benign tumours, central molecule in the top scoring network was vascular endothelial growth factor (VEGF). VEGF has been identified as a vascular permeability factor, angiogenic cytokine, and a survival factor in mammary tumours. VEGF overexpression and its role in angiogenesis of CMTs have been reported [77]. VEGF can act directly on T lymphocytes and elevated VEGF levels may contribute to the aberrant MMP-9 secretion by mammary tumour bearing T cells [78]. VEGF is known to stimulate IL-18 production, which in turn, promotes cancer cell migration, proliferation, angiogenesis and decreases cancer cell susceptibility to lymphocyte mediated cytotoxicity [79–80]. In this network, overexpression of Vasohibin 2 (VASH2) was also seen. VASH2 is a VASH1 homolog, expressed in mononuclear cells and has been reported as an angiogenesis stimulator in mice [81]. Higher expression of VASH2 induces expression of growth factors and promotes cell proliferation in breast cancer [82]. VASH2 is also involved in the proliferation of hepatic and ovarian cancers [83–84]. Another protein in our dataset lying within VEGF network is maternal embryonic leucine-zipper kinase (MELK), a highly conserved serine/threonine kinase that is essential for cell cycle regulation, organogenesis and stem cell proliferation [85]. Interestingly, MELK is additionally involved in the initiation and propagation of numerous human cancers and correlates with poor prognosis [86–87]. Expression of MELK has been reported in canine prostate carcinoma derived cell lines [88]. In this study we also found increased expression of MELK in CMTs. Further in this network, up-regulation of ST8SIA4 was seen to be directly linked to VEGF. ST8SIA4 is involved in sialylation of proteins associated with cancer progression. ST8SIA4 is significantly up-regulated in breast cancers and its up-regulation is highly correlated with cancer malignancy [89–90]. Dual specificity phosphatase (DUSP6), is another protein within the VEGF network which was found to be overexpressed in CMTs in the present study. DUSP6 expression has been reported to play an oncogenic role in breast cancers [91]. Moreover, DUSP6 is part of a high-risk gene signature for non-small cell lung cancer [92], and its expression is significantly correlated with high extracellular signal-regulated kinase (ERK) 1/2 activity in primary human ovarian cancer cells [93]. Other proteins in the VEGF network, whose overexpression was observed, were cyclin-dependent kinase inhibitor-3(CDKN3) and PROM1 (CD133). CDKN3 is a dual-specificity protein tyrosine phosphatase of the CDC14 group, which is often overexpressed in human cancers [94–95]. Since rapidly growing tumours have more mitotic cells, the high level of CDKN3 in mitotic phase provides the best plausible explanation for the frequent CDKN3 overexpression in human cancers [96]. PROM1 is linked to VEGF directly in this network and is attributed to poor prognosis in triple negative breast cancer due to its nuclear mislocalization [97]. Up-regulation of PROM1 increases the invasive capability, metastasis and drug-resistance of breast cancers [98].

### Functional significance of differentially up-regulated proteins in CMTs

Proteomic analysis of malignant CMT and healthy mammary tissues, revealed seven proteins highly up-regulated in CMTs, namely ANXA2, APOCII, CDK6, GATC, GDI2, GNAQ and MYH9. Overexpression of ANXA2 and CDK6 has previously been reported in canine gliomas [99] and CMT cell lines [100]. GNAQ is implicated in canine melanomas and T cell lymphomas by different researchers [101–102]. To the best of our knowledge, none of the other overexpressed proteins has previously been reported in cancers affecting dogs. However, role of these proteins in cancer pathogenesis in humans is well studied. Annexin A2 (ANXA2), a calcium-dependent phospholipid-binding protein localized at the extracellular surface of endothelial cells having role in regulation of cellular growth and in signal transduction pathways [103]. ANXA2 over expression has been reported in cancers of the breast, liver, prostate and pancreas, where it plays role in cancer cell migration, invasion, metastasis and adhesion [104–107]. Apolipoprotein C2 (APOCII) is a lipid binding protein belonging to the apolipoprotein gene family. This protein activates the enzyme lipoprotein lipase, which hydrolyzes triglycerides and thus provides free fatty acids for cells. APOC2 is one of the biomarkers that have been used in differentiating bladder cancer from hernia [108]. Cyclin-dependent kinase 6 (CDK6) plays a vital role in regulating the progression of the cell cycle. More recently, CDK6 has also been shown to have a transcriptional role in tumour angiogenesis, being a part of a transcription complex that induces the expression of the tumour suppressor p16^INK4a^ and the pro-angiogenic factor VEGF-A [109]. Emerging evidence suggests that certain tumour cells require CDK6 for proliferation [110]. Guanine nucleotide-binding protein G(q) subunit alpha (GNAQ) is **i**nvolved in glutamine metabolism and allows the formation of correctly charged Gln-tRNA(Gln). Researchers have demonstrated that glutamine is a major nutrient involved in multiple aspects of cancer metabolism [111]. Most cancer cells are dependent on glutamine and cannot survive in the absence of exogenous glutamine and targeting glutamine metabolism has been recently looked upon as a promising strategy for cancer treatment [112]. Rab GDP dissociation inhibitor beta (RABGDIB/GDI2) is a member of the GDP dissociation inhibitor family that controls the recycling of Rab GTPases involved in membrane trafficking. GDI2 has been proposed as a tumor suppressor gene and acts as an indicator of tumorigenesis in NSCLC [113]. Increased expression of GDI2 has been reported pancreatic carcinoma [114]. Guanine nucleotide-binding protein G(q) subunit alpha(*GNAQ*) encodes for alpha subunit of q class of heterotrimeric GTP binding protein (Gq) that mediates signals between G-protein-coupled receptors (GPCRs). Genetic, biochemical and biological analysis has shown that *GNAQ* behaves as a bona fide human oncogene. Recent studies have shown that G*NAQ* is a pivotal cancer gene in blue naevi [115]. Myosin Heavy Chain 9, (MYH9) encodes NM II-A protein which exists primarily in the cytoplasm and is involved in cytokinesis, cell motility and maintenance of cell shape. The role of MYH9 in cell migration, invasion, and metastasis has been established [116–117]. The overexpression of MYH9 is related to a poor prognosis in oesophageal, bladder, and gastric cancer [116–120].

Thus, the proteomic studies have identified seven proteins unregulated in CMTs. Overexpression of these proteins has not been reported previously in CMTs, however their role in human cancer pathogenesis is well studied. Thus, these proteins need to be studied further for their role in pathogenesis of different cancers of dog.

## Conclusions

This study presents gene expression profile of spontaneously occurring canine mammary tumours. Differential gene expression was analysed in context of gene networks and pathways to have insight into the signature pathways associated with benign and malignant CMTs. The research will also add to the knowledge of human cancer studies as dog mammary cancer is a useful model for human breast cancer studies. Presently, the genes accountable for the aggressive behaviour of mammary cancer are not very clear, and poor prognosis associated with malignant mammary cancers emphasize the necessity to unravel the underlying pathways and genes which could act as targets for therapy. In this study we have identified several genes which play a diverse role in tumour angiogenesis, cancer onset and pathogenesis. We have also identified important canonical pathways and key networks in CMTs. The identified differential genes need to be further validated as therapeutic targets and prognostic markers for mammary cancer. In future, further investigations are required to delineate the gene expression patterns among different types & grades of cancers and their response to specific therapies. Further research for identification of gene signatures associated with prediction of response to specific chemotherapies for developing personalized chemotherapeutic regimens is need of the hour.

## Supporting information

S1 Fig Canonical pathway—cyclins and cell cycle regulation in malignant (A) and benign (B) mammary tumour.
(TIF)

S2 Fig Canonical pathway—mitotic role of polo like kinases in malignant (a) and benign (b) mammary tumour.
(TIF)

S3 Fig Canonical pathway—Wnt/β-catenin signalling in malignant (A) and benign (B) mammary tumour.
(TIF)

S4 Fig Overlapping canonical pathways in malignant vs. healthy mammary tissues. (TIF)

S5 Fig Overlapping canonical pathways in benign vs. healthy mammary tissues. (TIF)

S1 Table Histopathological details of tumour tissues used for the study. (DOCX)

S2 Table Primer sequences used for qPCR of target genes. (DOCX)
