# Supplementary figures and images for: Gene expression profiling of spontaneously occurring canine mammary tumours: Insight into gene networks and pathways linked to cancer pathogenesis
Source: PLoS One. 2018 Dec 5;13(12):e0208656. doi: 10.1371/journal.pone.0208656 (PMC6281268; doi:10.1371/journal.pone.0208656)

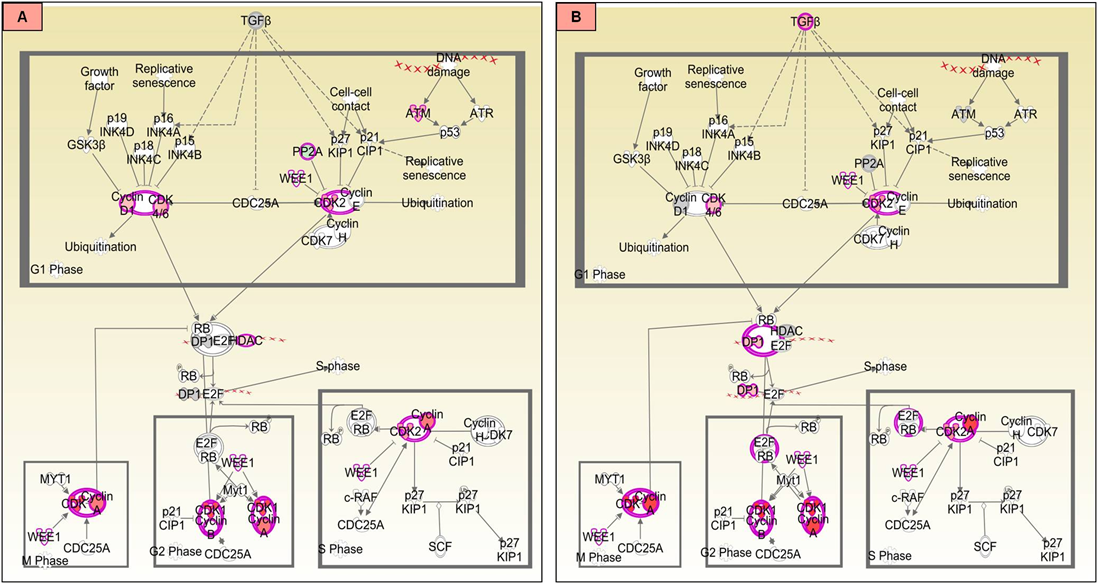

Supplement: S1 Fig — Canonical pathway—cyclins and cell cycle regulation in malignant (A) and benign (B) mammary tumour. (TIF) [file pone.0208656.s001.tif]

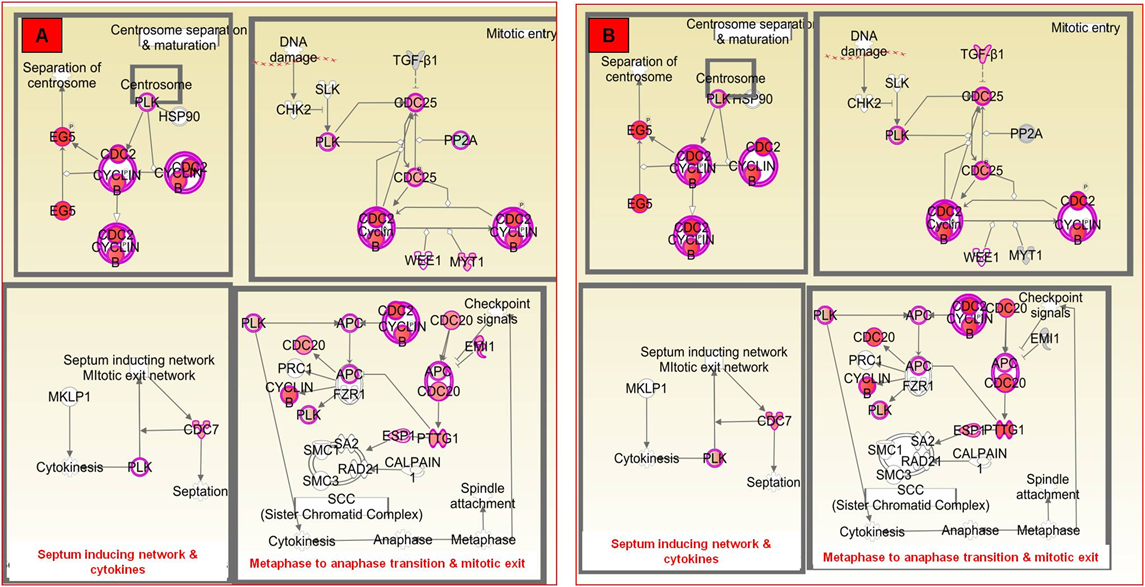

Supplement: S2 Fig — Canonical pathway—mitotic role of polo like kinases in malignant (a) and benign (b) mammary tumour. (TIF) [file pone.0208656.s002.tif]

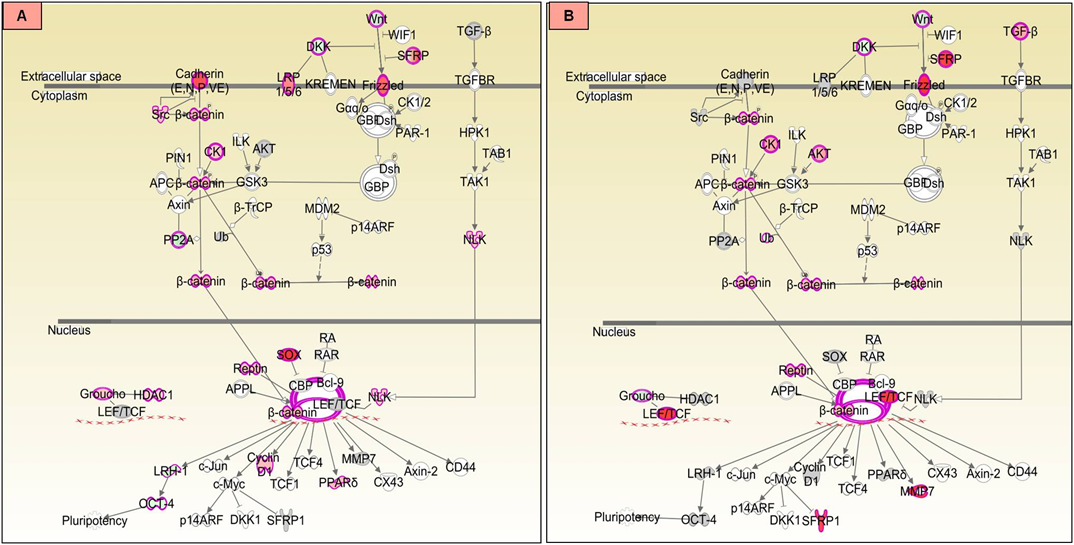

Supplement: S3 Fig — Canonical pathway—Wnt/β-catenin signalling in malignant (A) and benign (B) mammary tumour. (TIF) [file pone.0208656.s003.tif]

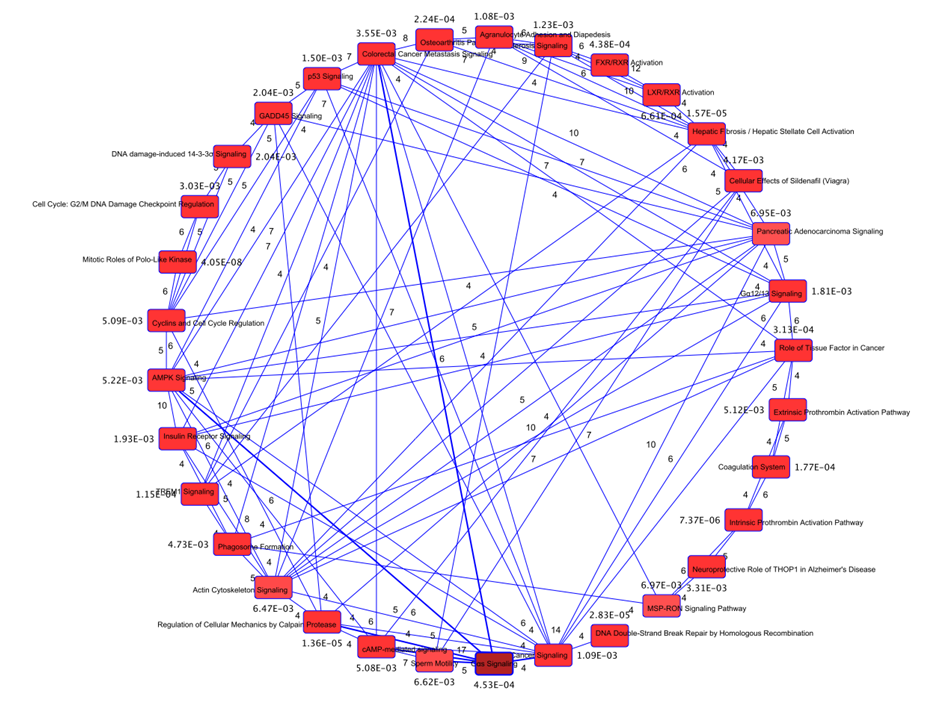

Supplement: S4 Fig — (TIF) [file pone.0208656.s004.tif]

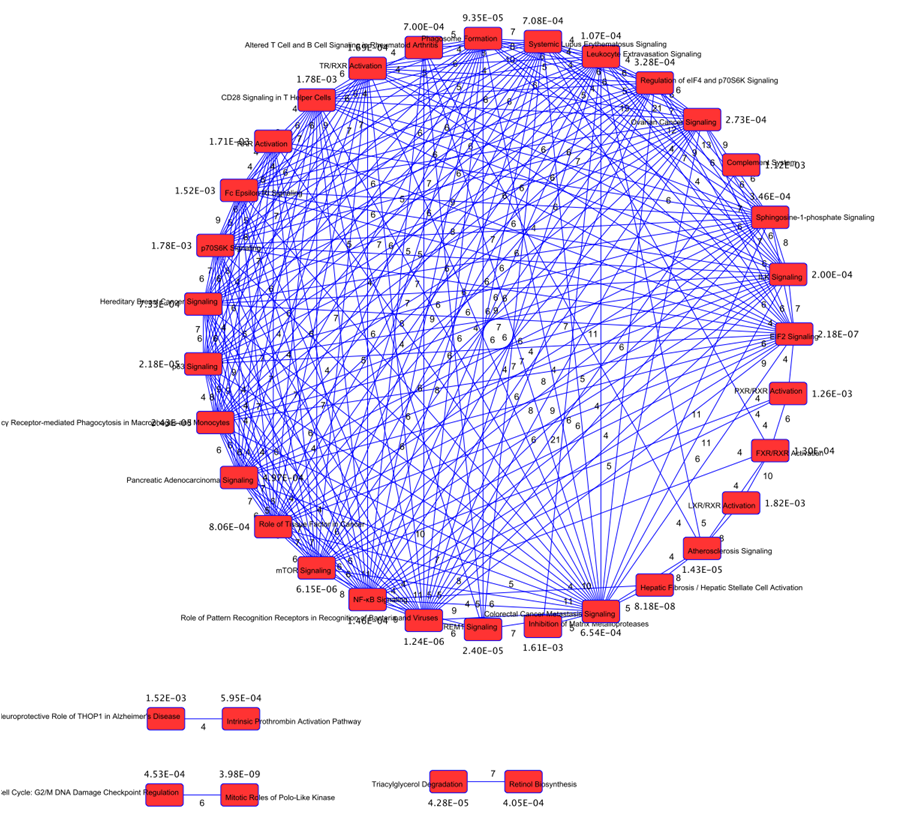

Supplement: S5 Fig — (TIF) [file pone.0208656.s005.tif]
