# Supplementary material for: Gene expression profiling of spontaneously occurring canine mammary tumours: Insight into gene networks and pathways linked to cancer pathogenesis
Source: PLoS One. 2018 Dec 5;13(12):e0208656. doi: 10.1371/journal.pone.0208656 (PMC6281268; doi:10.1371/journal.pone.0208656)
Supplement: S1 Table — (DOCX) [file pone.0208656.s006.docx]

**SUPPLEMENTARY TABLES:**

**S1 Table . Histopathological details of tumour tissues used for the study**

| **Code** | **Histopathological classification** | **Benign / malignant grade** |
| --- | --- | --- |
| BENIGN 1 | Adenoma 1 | Benign |
| BENIGN 2 | Adenoma 2 | Benign |
| BENIGN 3 | Papillary adenoma | Benign |
| BENIGN 4 | Cystadenomyoepithelioma | Benign |
| HEALTHY 2 | Healthy tissue | Normal mammary gland |
| HEALTHY 3 | Healthy tissue | Normal mammary gland |
| HEALTHY 1 | Healthy tissue | Normal mammary gland |
| MALIGNANT 1 | Malignant , II | Tubuloacinar solid carcinoma |
| MALIGNANT 2 | Malignant, II | Papillary and squamous mixed |
| MALIGNANT 3 | Malignant, II | Myoepithelioma |
| MALIGNANT 4 | Malignant, III | Squamous cell carcinoma |
| MALIGNANT 5 | Malignant, II | Lipid rich carcinoma |
| MALIGNANT 6 | Malignant, I | Fibrosarcoma |
