# Supplementary material for: Gene expression profiling of spontaneously occurring canine mammary tumours: Insight into gene networks and pathways linked to cancer pathogenesis
Source: PLoS One. 2018 Dec 5;13(12):e0208656. doi: 10.1371/journal.pone.0208656 (PMC6281268; doi:10.1371/journal.pone.0208656)
Supplement: S2 Table — (DOCX) [file pone.0208656.s007.docx]

**S2 Table. Primer sequences used for qPCR of target genes**

| **Gene Name** | **Primer** | **Sequence** |
| --- | --- | --- |
| COL11A1 | Forward | GGAGAAAAGGGACAGAAAGGAG |
|  | Reverse | GACCCATAAGACCCGCAG |
| SFRP2 | Forward | GATGACAACGACATAATGGAAACTC |
|  | Reverse | CCTTTCAGACACACCGTTCAG |
| K1F11 | Forward | AACAAAGAAGAGATCAGTCAGGAC |
|  | Reverse | TGACTTTTTATGCTGGAAAAACGG |
| RAB31 | Forward | GCTGAATCCATAGGTGCTGTTG |
|  | Reverse | TTCCCCAGTTTGATTGCTCC |
| CDCA3 | Forward | ATCTCCCCTCACCATCCTAC |
|  | Reverse | GCCTCCAGTTTTCAGAAGTCG |
| CPAK2L | Forward | GCAACACCCCAAATAACCAAG |
|  | Reverse | GCCACGACTATATCATGTTCCTG |
| MMP9 | Forward | CGC ATG ACA TCT TCC AGT ACCA |
|  | Reverse | CCG AGA ATT CAC ACG CCA GTA |
| TLR2 | Forward | AAT CCC CCG TTC AAG TTG TG |
|  | Reverse | ATG GTT TTG CGG CTC TTC TC |
| CTSS | Forward | TCT GGG AGA CAT GAC TGG TGA A |
|  | Reverse | AAG TGA CAT TTC TCT GCC ATT GG |
| BIRC5 | Forward | GCC CAG TGT TTC TTCTGC TT |
|  | Reverse | CCA GAT GAA TGT TTT TTA TGC |
| CHI3L1 | Forward | CTCTATGACACGCTGAACACA |
|  | Reverse | TGGGTCTTGGAGGCTATTTTG |
| TOP2A | Forward | TGTGGAAAGAAGACTTGGCTAC |
|  | Reverse | CGCAAGGAGAAGGCAAAAC |
| PGAM1 | Forward | GACCATCCCTTCTACAGCAAC |
|  | Reverse | GGCAATGGTATCCTTCAGACTC |
| BIRC2 | Forward | AGG CGT CCC CGTGTC CGAGAG |
|  | Reverse | TAG CAT CAG GCCGCAGCA GAA GC |
